# Amylin exacerbates tau pathology in the visual cortex of diabetic mice by impairing lysosomal activity

**DOI:** 10.1016/j.gendis.2025.101602

**Published:** 2025-03-18

**Authors:** Daniel Moreira-Silva, Melike Yuksel, Moorthi Ponnusamy, Mitchell T. Hansen, Joseph D. McMillan, Sneha Geethakrishnan, Shuai Wang, Lisa A. Collier, Gopal Thinakaran

**Affiliations:** a USF Health Byrd Alzheimer's Center and Research Institute, University of South Florida, Tampa, FL 33613, USA; b Department of Molecular Medicine, USF Morsani College of Medicine, University of South Florida, Tampa, FL 33612, USA

**Keywords:** Alzheimer's disease, Diabetes, Lysosomal dysfunction, PS19 mice, Streptozotocin, Tauopathy

## Abstract

The aggregation of the peptide hormone amylin in the pancreas is a pathological hallmark of type-2 diabetes. Additionally, amylin can form aggregates in the brain, promoting β-amyloid deposition and tau phosphorylation in Alzheimer's disease. The cross-seeding between amylin and tau exacerbates tau pathology spread and synaptic loss, leading to neurodegeneration and cognitive deficits. Given the link between lysosomal dysfunction and tauopathy in the brain and amylin aggregation in the pancreas, we hypothesized that amylin could potentially worsen tau pathology in diabetic mice. We administered streptozotocin and/or amylin peripherally to the PS19 model of tauopathy at 3 months and characterized them at 6 months of age. We found that streptozotocin diminished body weight gain, increased blood glucose levels, worsened motor performance, and improved fear-conditioned memory in PS19 mice. Both amylin and streptozotocin administration prompted the emergence of tau pathology in the pancreas, which coincided with a decrease in the number of lysosomes in pancreatic islets. Mice treated with amylin and streptozotocin also developed robust tau pathology concomitant with lowering lysosomal cathepsin D levels in the visual cortex. These findings suggest that in diabetic mice, amylin administration diminished pancreatic lysosomes, possibly increasing the number of amylin aggregates that reached the brain and contributing to the worsening of tau pathology due to lysosomal impairment in the visual cortex. The outcome of our research enhances the understanding of the cellular pathways by which amylin may serve as a link between the pancreas-brain axis during diabetes, influencing the risk of developing tau pathology.

## Introduction

The current estimated global prevalence of diabetes is approximately 10%,^1^ and the incidence of diabetes is linked to a 65% increased risk of Alzheimer's disease (AD).^2^ Recent evidence has led to the emergence of the concept that AD represents a brain type of diabetes, also referred to as type 3 diabetes, since AD and diabetes share many pathological features, such as insulin resistance, memory impairment, neuroinflammation, and protein misfolding.^3^, ^4^, ^5^ From this perspective, there is a growing interest in investigating the roles of β-amyloid, tau, and amylin in the brain and peripheral organs, as well as potential cross-seeding mechanisms between the protein aggregates.^3^^,^^6^^,^^7^

The aggregation of toxic amylin oligomers in the pancreas is one of the pathological hallmarks of diabetes identified 110 years ago by Opie.^8^ Amylin (also known as islet amyloid polypeptide) is a 37-amino-acid peptide produced and secreted by the pancreas that has hormonal functions. It can cross the blood-brain barrier and activate receptors in the brainstem, suppressing glucagon release, gastric emptying, and food intake, leading to an acute decrease in blood glucose levels and a chronic reduction in body weight.^9^^,^^10^ Moreover, amylin oligomers participate in a cross-seeding interaction with β-amyloid peptides, which accumulate in brain senile plaques, a hallmark of AD.^11^, ^12^, ^13^ Like β-amyloid, amylin aggregates trigger several AD-related pathological mechanisms, including mitochondrial dysfunction,^13^ neuroinflammation, and cognitive deficits.^14^

The presence of phosphorylated tau (p-tau) protein aggregates in the brain is another characteristic pathological hallmark of AD, widely used as a criterion to distinguish disease progression (Braak stages).^15^ The accumulation of p-tau disrupts microtubule stabilization, leading to the formation of neurofibrillary tangles, which is closely linked to synaptic dysfunction, memory impairments, and cell death in AD and diabetes.^16^, ^17^, ^18^, ^19^, ^20^ Evidence suggests that amylin can interact with tau protein and potentially worsen tau pathology.^21^, ^22^, ^23^, ^24^ Amylin formed hetero-oligomers with an amyloidogenic cytotoxic tau fragment *in vitro*,^23^ and administering amylin increased p-tau levels in primary neurons in a dose-dependent manner.^25^ Recent studies confirm that amylin also cross-seeds with p-tau *in vivo*, promoting the spread of tau pathology in the brain and worsening synapse loss and cognitive deficits in the PS19 P301S human tau transgenic line.^21^

The failure of proteolytic and autophagic mechanisms has been identified as one of the main reasons for the accumulation of tau^26^ and other protein aggregates, such as β-amyloid^27^ and amylin.^28^, ^29^, ^30^ In AD tau pathology, the reduced efficiency in the clearance of p-tau within autophagic vacuoles disrupts neuronal communication, leading to synaptic toxicity and, ultimately, cell death.^31^^,^^32^ Alterations in the activity of lysosomal enzymes contribute to the accumulation of misfolded proteins in AD and other neurodegenerative diseases.^33^, ^34^, ^35^ Variants in cathepsin D (CatD), a lysosomal peptidase that plays a role in degrading many aggregation-prone protein substrates, including amyloid and tau, are associated with an increased risk for AD,^35^, ^36^, ^37^, ^38^, ^39^, ^40^, ^41^ and higher levels of cathepsin B have been observed in a triple transgenic model of AD pathogenesis, known as 3xTg-AD mice.^42^ Additionally, lysosomal-associated membrane protein 1 (LAMP-1) levels were found to be elevated in the brain cortex of patients with early-onset familial AD, and CatD was diffusely distributed in the cytoplasm, suggesting damage to the lysosomal integrity.^26^

Failures in autophagic mechanisms have also been linked to diabetes-related chronic complications. The hyperglycemic state induced by diabetes down-regulates the autophagy-related gene expression and correlates with the incidence of retinopathy, nephropathy, and cardiopathy.^43^ Interestingly, the impairment of lysosomal functions in diabetes seems to be associated with the presence of amylin oligomers. Overexpression of amylin leads to impaired autophagy, disrupting lysosomal clearance *in vivo* and promoting oxidative damage and apoptosis of pancreatic β-cells.^28^^,^^29^ Although it has been reported that amylin can affect tau pathology in an AD mouse model, the cellular mechanisms underlying the interaction between tau and amylin have not been thoroughly investigated. It remains unclear whether amylin alone is sufficient to mimic diabetes's potential to worsen tau pathology. Understanding the interaction between amylin and tau pathology is crucial in uncovering how diabetes can influence the risk of developing AD.

Our study utilized the intraperitoneal administration of streptozotocin (STZ) as a model for diabetes. The STZ model is founded on pancreatic β-cell death and replicates many of the features observed in both AD and diabetes: cognitive decline, protein aggregation (p-tau, β-amyloid, and amylin), neuroinflammation, and changes in the insulin receptor cascade.^20^^,^^44^^,^^45^ It is known that diabetes worsens AD symptoms in humans and rodent models. However, the impact of peripheral STZ administration on tau pathophysiology has not been examined in the PS19 model. Thus, we explored whether diabetic conditions induced by STZ would worsen tau pathophysiology and investigated the cellular pathways through which amylin could synergize with diabetes to exacerbate tau pathology. Our results show that amylin synergized with diabetes, worsening tau pathology by impairing lysosomal activity in the visual cortex.

## Materials and methods

### Animals

All experimental procedures related to animal care and experimental manipulation were carried out in agreement with the Institutional Animal Care and Use Committee policies at the University of South Florida, Tampa. PS19 transgenic mice (B6.Cg-Tg(Prnp-MAPT∗P301S)PS19Vle/J; Jax#024841) were purchased from The Jackson Laboratory and maintained in the C57BL/6J background. The PS19 line expresses P301S mutant human tau driven by the mouse prion protein promoter, leading to the development of tau pathology in an age-dependent manner.^46^ Mice were maintained at 22 ± 2 °C, under a 12 h/12 h light/dark cycle, and had free access to food and water. Only male mice were used because female rodents take longer to develop amylin aggregates in the brain than males,^47^ and there is a tremendous sex difference in the extent of tauopathy between males and females in the PS19 mouse model.^48^^,^^49^ The number of animals used per group is indicated in the figure legends for each experiment.

### Treatments and experimental design

Three-month-old male PS19 mice received intraperitoneal injections of STZ (60 mg/kg) or citrate buffer (CIT, pH 4.5) for 5 days. After 45 days, the mice received daily intraperitoneal injections of amylin (AMY, 2 mg/kg) or phosphate-buffered saline (PBS). The experimental groups are referred to as CIT/PBS, STZ/PBS, CIT/AMY, and STZ/AMY. To prepare amylin aggregates, synthetic amylin peptide (AS-60804, AnaSpec) was dissolved in PBS at a concentration of 50 μM and maintained at 37 °C for 72 h with intermittent shaking. Administering amylin aggregates at this concentration is sufficient to increase blood amylin levels that equal or exceed levels found in diabetic animals.^47^ The body weight and blood glucose levels of animals were monitored regularly. To measure fasting glucose levels, mice fasted for 4 h before blood collection. Blood samples were drawn from the facial vein using an 18G needle, and glucose blood levels were measured using Bayer Contour One Next glucometer.

**Figure 1:**
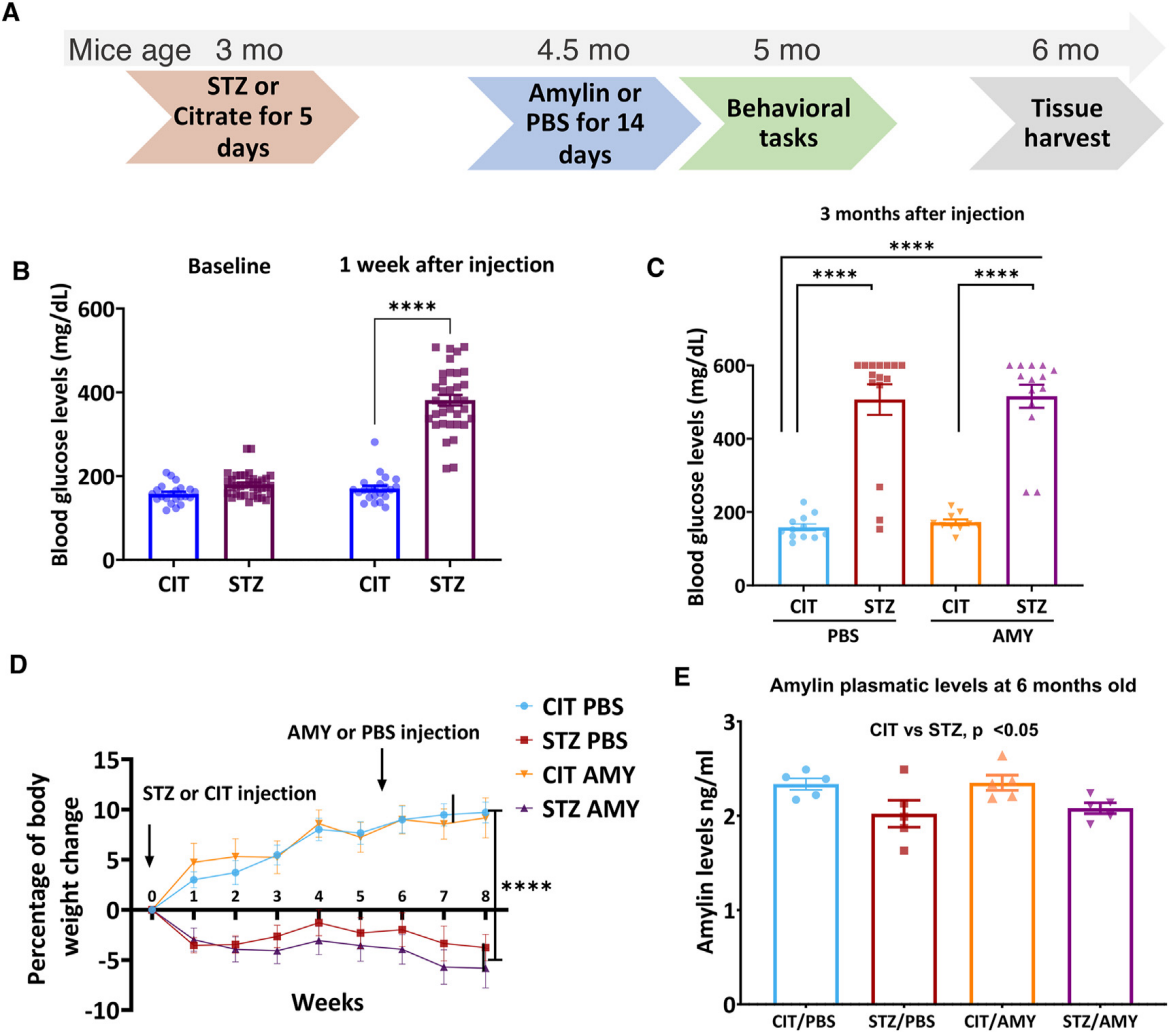
The experimental timeline and physiological parameters measured in PS19 mice. **(A)** Three-month-old PS19 mice were administered with CIT or STZ and PBS or AMY. The timeline of the experiment is represented in the schematics. **(B)** One week after being administered with STZ (60 mg/kg), PS19 mice presented significantly higher blood glucose levels (*P* < 0.0001; CIT, *n* = 25; STZ, *n* = 36). **(C)** CIT and STZ groups were equally divided for AMY (2 mg/kg) or PBS injections at 4.5 months of age. Amylin administration did not affect the differences between the groups. The blood glucose in STZ/PBS and STZ/AMY groups remained at equally higher levels as compared with CIT-injected mice (*P* < 0.0001; CIT/PBS, *n* = 11; STZ/PBS, *n* = 15; CIT/AMY, *n* = 10; STZ/AMY, *n* = 14). **(D)** STZ/PBS and STZ/AMY mice presented a lower rate of body weight gain than CIT/PBS and CIT/AMY (*P* < 0.0001; CIT/PBS, *n* = 12; STZ/PBS, *n* = 16; CIT/AMY, *n* = 10; CIT/AMY, *n* = 17). **(E)** The levels of plasmatic amylin measured by ELISA in samples collected from 6-month-old PS19 mice were lower in STZ-injected mice (*P* < 0.05 for diabetes factor in Two-way ANOVA) (CIT/PBS, *n* = 6; STZ/PBS, *n* = 5; CIT/AMY, *n* = 5; CIT/AMY, *n* = 5).

At 5 months of age, the mice underwent behavioral tests to evaluate their motor and cognitive performance. After the behavioral analyses were concluded, the animals were euthanized at approximately 6 months of age, and their blood, pancreas, and brain were harvested for further analysis with immunostaining and enzyme-linked immunosorbent assay (ELISA) (Fig. 1A).

### Behavioral analyses

The mice underwent the following behavioral tests to evaluate their cognitive and motor performance. Experiments were recorded by an overhead camera, and the animal's movements during all the tasks were analyzed by Ethovision software (Noldus). Non-transgenic mice were used as positive controls of the behavioral tasks (data not shown).

Open field and novel object recognition tests were conducted in an open field square arena (white plastic box, 23 cm × 23 cm). During habituation, the animals were allowed to explore the arena freely for 8 min. The distance traveled and time spent in the center and periphery of the arena were recorded as anxiety-like and motor parameters. 24 h after habituation, the mice were allowed to explore the arena for 5 min in the presence of two identical objects (objects A and B) placed on opposite sides of the arena 3 cm from the walls (training phase). The next day, the mice were allowed to explore the arena for 5 min in the presence of one familiar (object B) and a novel object (object C) to evaluate long-term recognition memory (test phase). The objects used in the task were similar in volume but different in shape and color. The time spent exploring each object was recorded, and the discrimination index was calculated using the formula: discrimination index = the time exploring object C/(the time exploring object B + the time exploring object C).

The elevated plus maze test was carried out in a "+"-shaped apparatus made of Plexiglass with a central area, two oppositely positioned closed arms, and two oppositely positioned open arms. The apparatus was elevated above the floor. The mice were allowed to explore the elevated plus maze for 8 min. This test evaluates locomotion and anxiety, measuring parameters such as the time spent in the open arms and the total distance traveled.

The composite phenotype scoring is a protocol for quantifying coordination, motor, and gait impairments in mouse models of neurodegeneration. Composite phenotype scoring includes the ledge test, hind limb clasping, gait, and kyphosis.^50^ The measures were scored with a scale in which 0 represents an absence of impairments in the behavior observed, and 3 represents the most severe expression of the phenotype. The ledge test measured the coordination of the mice while they walked along the cage's ledge. Hindlimb clasping evaluated ataxia by observing the hindlimbs' position when mice were lifted by the tail. The retraction of hindlimbs is normally related to cerebellar ataxia, whereas hindlimbs are splayed outward to indicate the absence of ataxia. The gait was analyzed by watching the way the mice moved, supporting their weight over the limbs and positioning their abdomen above the ground. The presence of kyphosis, a dorsal curvature manifested in several neurodegenerative diseases, was investigated by observing how the mice walked and straightened their spine.

The spontaneous alternation in the Y-maze was evaluated to analyze the working memory performance. The task was carried out in a Y-shaped maze with three equal opaque plastic arms at a 120° angle from each other. An alternation was defined as three consecutive entries into three different arms. The percentage of alternations was calculated by the following equation: percentage of alternations = number of alternations observed/maximum number of possible alternations × 100.

The Morris water maze test was performed in a room with diffuse lighting and several visual cues placed on the walls. The pool (a 122 cm tank) was filled with water at room temperature (22 ± 1 °C), and white dye was added to make the water opaque during all the phases of the Morris water maze test. A circular platform (12 cm in diameter) was placed and submerged approximately 1 cm below the water surface in the tank. On the first day, mice were allowed to stay for 30 s on the platform to learn its location and the environmental context around it. The mice then underwent four consecutive days of training, where each mouse performed two training sessions. Each session consisted of two trials of 60 s with a 30-s interval between the trials. During the trial, the mouse was released in the pool facing the wall, in a randomly selected quadrant, and allowed to swim freely for 60 s to find the platform. When the mice found the platform, they were allowed to rest on it for 30 s. If unsuccessful, they were gently guided to the platform and rested for 30 s. The second session was performed after all the mice had finished the first session. The probe test was carried out 24 h after the end of the training phase. The platform was removed, and each mouse was released into the pool to search for it for 60 s. The latency to find the platform during the training phase, the time spent in the platform quadrant in the probe test, and motor parameters throughout all the phases were measured for further analysis.

Contextual fear conditioning was performed to evaluate hippocampal-dependent emotional memory^51^ using an Actimetrics fear conditioning chamber and Coulborn Instrument shocker. The mouse movements were tracked and recorded using FreezeFrame 4 software. On the training day, each mouse was placed in the chamber for 7 min. After the first 2 min, a brief footshock (0.5 mA, 2 s) was delivered by the grid floor, and after a 2 min interval, the same shock stimulus was presented to strengthen the association. The mouse remained in the chamber for 3 min before returning to the home cage. After 24 h, each mouse was placed in the chamber for 5 min without receiving any stimulus to test the retention of the contextual fear-conditioned memory.

### Euthanasia, harvest, and preparation of tissue

Mice were deeply anesthetized with isoflurane, and approximately 1 mL of blood was collected through a cardiac puncture in EDTA tubes. The blood samples were centrifuged at 1000 *g* for 10 min, and the supernatant containing the plasma was transferred to fresh tubes and stored at −80 °C. After the exsanguination, the animals were transcardially perfused with PBS (pH 7.4). At the end of perfusion, the brains and pancreas were removed, post-fixed for 24 h in 4% paraformaldehyde solution, stored in 70% ethanol solution, and later embedded in paraffin. The paraffin blocks were cut into 5-μm-thickness sagittal sections on a microtome. The sections were deparaffinized through sequential washes in xylene and rehydrated by immersing them in graded ethanol baths and water.

### Amylin ELISA

The protocol was followed according to the instructions of the manufacturer (RayBioTech). Briefly, the plate was equilibrated to room temperature, and diluted anti-amylin antibody was added to each well and incubated overnight. After washing, the standards, positive controls (containing biotinylated amylin), or samples were added to each well and incubated at room temperature with gentle shaking for 2.5 h. After washing, horseradish peroxidase-conjugated streptavidin solution was added, and the plates were incubated for 45 min with gentle shaking. After another round of washes, TMB One-Step Substrate Reagent was added to each well and incubated for 30 min with gentle shaking. Later, the stop solution was added, and the plates were read at 450 nm.

### Immunofluorescence staining and analysis

Multiplex immunostaining was performed on the intelliPATH FLX automated slide-staining system (Biocare Medical) as described,^49^ utilizing the manufacturer's reagents. Briefly, the primary and Alexa Fluor-conjugated secondary antibodies (Table S1) were diluted in Da Vinci reagent, and nuclei were stained with Hoechst. The slides were subject to epitope retrieval and sequentially incubated with each primary and secondary antibody for 1 h. The sections were dried at room temperature and mounted using Vectashield mounting medium (Vector Labs).

Stained sections were imaged on an automated Nikon Ti2 microscope fitted with a Yokogawa spinning disk field scanning confocal system. Images were acquired using a 4 × , 10 × , 20 × , or 60 × objective, and SoRa imaging with a 60 × objective combined with 4 × magnification was used for subcellular analyses. High-magnification z-stack images were converted to maximum intensity 2D projections and deconvolved in NIS-Elements software (Nikon).^52^ The immunostaining images were processed and quantified using QuPath and Fiji software, with multiple anatomical planes represented in different sections per mouse. For p-tau quantification, the total area of p-tau-positive staining was measured across various regions in four sections for each animal (the animal numbers are listed under each figure legend). These areas were summed (integrating the positive staining across sections, thus improving the precision of analysis) to calculate the p-tau-positive area ratio relative to the total area. The quantitative analysis of SoRa imaging was performed by collecting multiple randomly selected fields to measure the staining accurately.

### Statistical analysis

The statistical significance of physiological, behavioral, molecular, and pathological data was analyzed by two-way analysis of variance (ANOVA) followed by post hoc Šídák's multiple comparisons test using GraphPad Prism (v 9.0.1) and IBM SPSS Statistics (v 29.0.2.0) software. Diabetes (CIT *vs*. STZ comparison) and amylin (PBS *vs*. AMY comparison) were considered the categorical variables. For two-way repeated measure ANOVAs, time represents the within-subject factor. The number of experimental animals ranged from 9 to 15 per group for behavioral and physiological measurements, whereas 5 to 8 animals per group were used for histological and molecular analyses. Statistical significance was determined at *P*-values less than 0.05. Each graph plotted the mean ± standard error of the mean, with significant differences occurring when *P* was less than 0.05. Simple linear regression was used for correlational analysis, and the individual values were plotted for each experimental sample. If *P* < 0.05, the slope was considered different from zero and indicated a rate of change in the dependent variable (*e.g.*, behavior) per unit change in the independent variable (*e.g.*, pathology marker). The significance levels for all the statistical analyses are as follows: ∗*P* < 0.05, ∗∗*P* < 0.01, ∗∗∗*P* < 0.001, and ∗∗∗∗*P* < 0.0001.

## Results

### The administration of STZ raises blood glucose levels and hinders weight gain in PS19 mice

Our study was designed to investigate whether amylin would elevate the tau burden in diabetic mice in the PS19 human tau transgenic background. We administered a low dose of STZ for 5 consecutive days (an approach increasingly utilized as an animal model for diabetes) to PS19 mice at 3 months of age, well before overt tau pathology manifests in this model. The blood glucose levels and body weight of the mice were monitored to confirm the presence of diabetic symptoms and the reliability of the model. One week after STZ or CIT administration, the STZ-injected mice already demonstrated a significant increase in blood glucose levels (two-way ANOVA: diabetes factor, F_(1, 46)_ = 116.9, *P* *<* 0.0001; followed by Šídák's test: CIT *vs*. STZ, *P* *<* 0.0001) (Fig. 1B). At 6 months of age, *i.e.*, 3 months after STZ administration, blood glucose levels further increased (381 mg/dL at 1 week *vs*. above 500 ng/dL at 3 months). However, there was no interaction between diabetes and amylin factors, as the blood glucose levels in mice administered with STZ and amylin (STZ/AMY group) were not significantly different from those receiving STZ alone (STZ/PBS group) (Fig. 1C). Both STZ/PBS and STZ/AMY groups also failed to gain weight (repeated measures two-way ANOVA: diabetes factor, F_(1, 46)_ = 59.932, *P* *<* 0.0001; followed by Šídák's test: STZ/PBS and STZ/AMY *vs*. CIT/PBS and CIT/AMY, *P* *<* 0.01 in multiple time points). In contrast, CIT/PBS and CIT/AMY groups gained 5%–10% of their body weight 8 weeks after STZ or CIT injections (Fig. 1D). Finally, at 6 months of age, plasma amylin levels were lower in STZ-injected mice (two-way ANOVA: diabetes factor, F_(1, 16)_ = 10.08, *P* *<* 0.01) but the amylin factor did not have a significant effect and post-hoc tests did not reveal significant inter-group differences (Fig. 1E).

### The induction of diabetes worsened motor coordination and enhanced fear memory in PS19 mice

**Figure 2:**
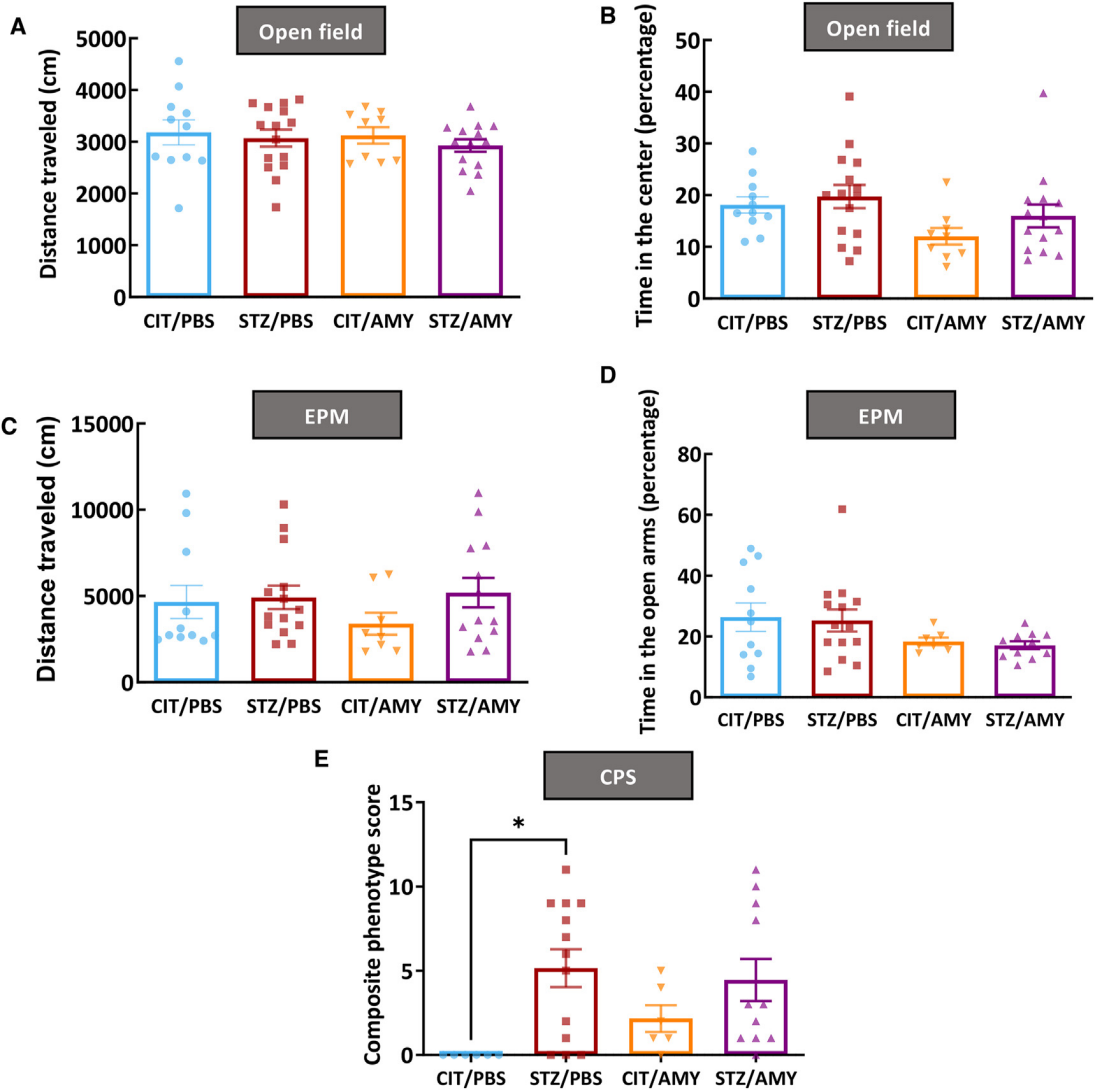
Motor and anxiety behaviors of PS19 mice injected with CIT or STZ and PBS or AMY. **(A**–**D)** After the end of treatments, PS19 mice did not present differences in the distance traveled (A) or time in the center of the open field (B) (CIT/PBS, *n* = 11; STZ/PBS, *n* = 15; CIT/AMY, *n* = 9; CIT/AMY, *n* = 14). Similarly, no differences were detected in the distance traveled (C) or time in the open arms of the elevated plus maze (EPM) (D) (CIT/PBS, *n* = 11; STZ/PBS, *n* = 14; CIT/AMY, *n* = 8; CIT/AMY, *n* = 13). **(E)** STZ/PBS presented a similar composite phenotype score (CPS) in comparison with STZ/AMY, but it was higher in relation to CIT/PBS, indicating that STZ impaired the motor coordination in PS19 mice (*P* < 0.05; CIT/PBS, *n* = 6; STZ/PBS, *n* = 13; CIT/AMY, *n* = 6; CIT/AMY, *n* = 11).

We conducted behavior tests at 6 months of age to ascertain the motor and cognitive performance of mice administered with STZ and/or amylin. Neither STZ nor amylin affected motor performance and anxiety behaviors in the open field test (Fig. 2A, B) and elevated plus maze (Fig. 2C, D). However, STZ-injected mice showed a higher composite phenotype score, which combined results from the ledge test, hind limb clasping, gait, and kyphosis performance (two-way ANOVA: diabetes factor, F_(1, 32)_ = 9.084, *P* *<* 0.001; followed by Šídák's test: STZ/PBS *vs*. CIT/PBS, *P <* 0.05, STZ/AMY *vs*. CIT/PBS, *P =* 0.08) (Fig. 2E). This finding indicated that STZ administration impaired motor coordination in PS19 mice at 6 months of age compared with littermates administered with CIT/PBS.

**Figure 3:**
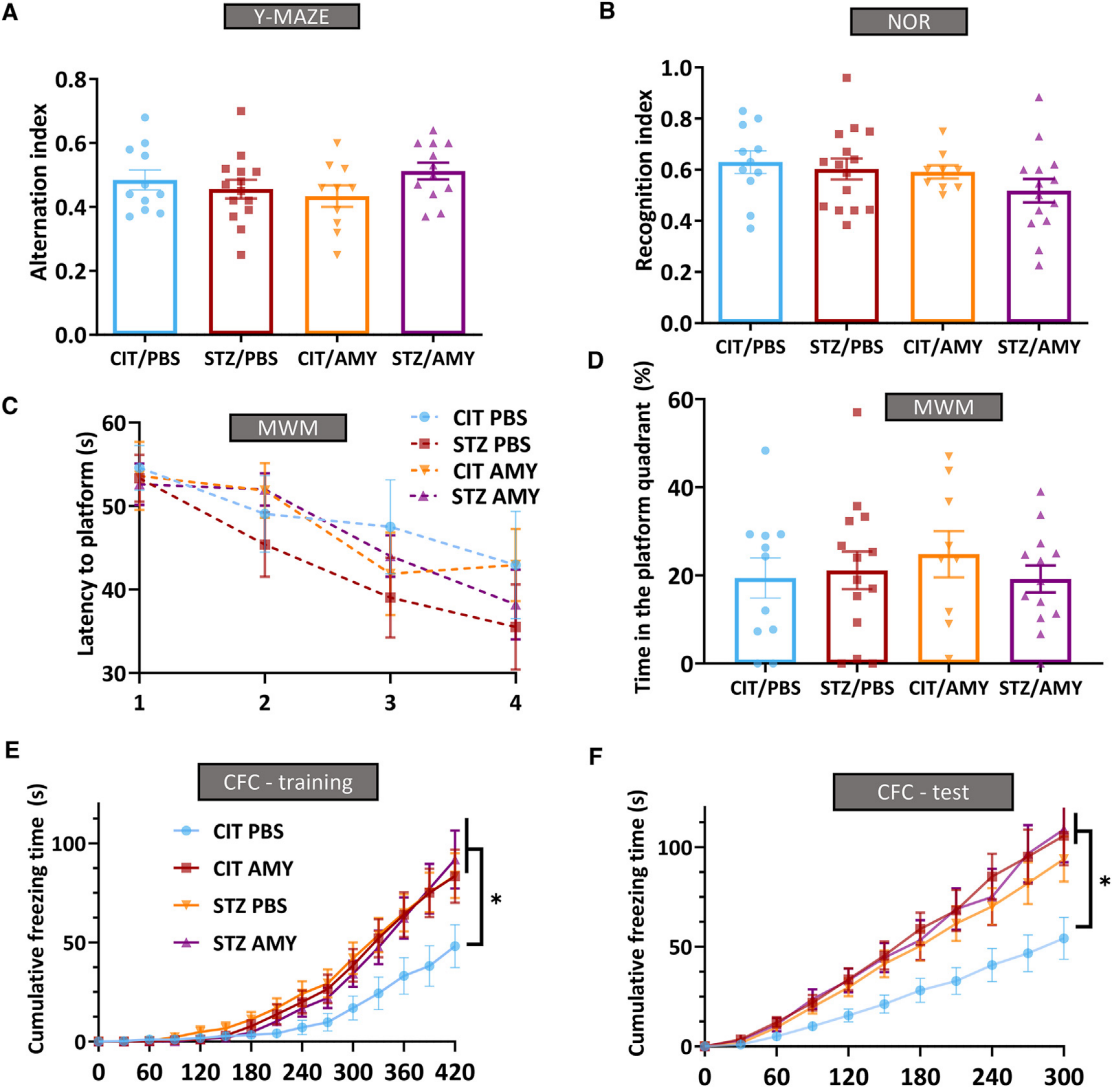
Cognitive performance of PS19 mice injected with CIT or STZ and PBS or AMY. **(A**–**D)** After the end of treatments, PS19 mice did not present differences in the working memory (Y-maze test) (A) (CIT/PBS, *n* = 11; STZ/PBS, *n* = 14; CIT/AMY, *n* = 10; CIT/AMY, *n* = 12), recognition memory in novel object recognition test (NOR) (B) (CIT/PBS, *n* = 11; STZ/PBS, *n* = 14; CIT/AMY, *n* = 9; CIT/AMY, *n* = 14), and spatial memory in the Morris water maze test (MWM) (C, D) (CIT/PBS, *n* = 10; STZ/PBS, *n* = 14; CIT/AMY, *n* = 9; CIT/AMY, *n* = 13). **(E, F)** STZ/PBS and STZ/AMY had a better performance in the contextual fear conditioning test (CFC) in comparison with CIT/PBS and CIT/AMY in the training and test sessions (*P* < 0.05; CIT/PBS, *n* = 10; STZ/PBS, *n* = 14; CIT/AMY, *n* = 9; CIT/AMY, *n* = 13).

None of the groups displayed significant differences in their working memory (Fig. 3A) or recognition memory (Fig. 3B), as assessed by spontaneous alternation in the Y-maze test and object preference in the novel object recognition test, respectively. Similarly, the latency to locate the platform during training and the time spent in the target quadrant during the probe trial in the Morris water maze test were indistinguishable among the different groups (Fig. 3C, D). In the contextual fear conditioning test, STZ administration enhanced the retention of fear memory (repeated measures two-way ANOVA: diabetes factor, F_(1, 42)_ = 6.886, *P* *<* 0.01), whereas amylin had no effect (Fig. 3E, F). These results indicated that STZ administration-induced behavioral changes were typically observed in diabetes models. In contrast, amylin did not impact mouse behavior directly or synergize with STZ.

### Peripheral amylin synergized with STZ, elevating p-tau levels in the visual cortex of PS19 mice

**Figure 4:**
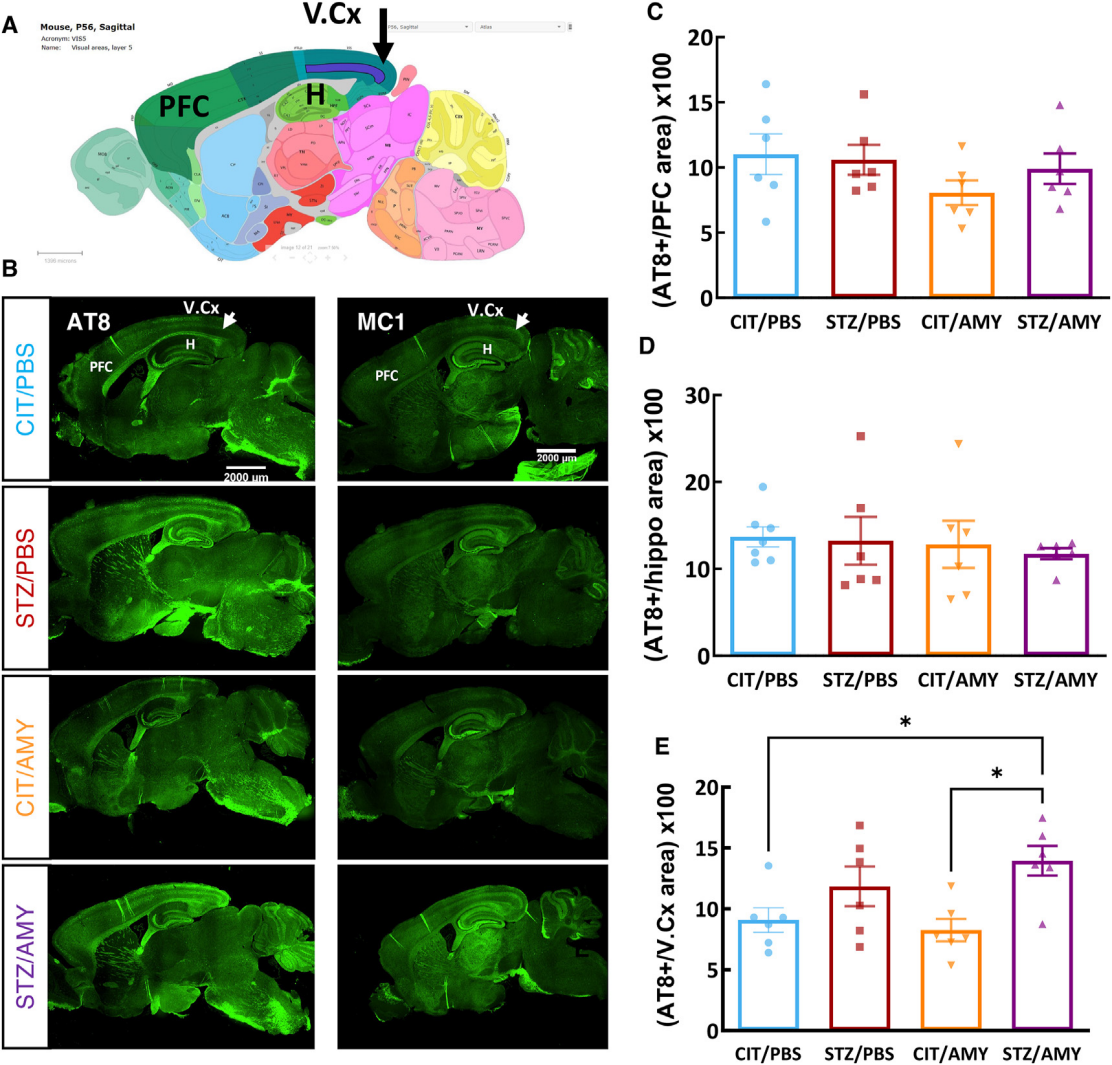
Immunostaining for p-tau in the brains of PS19 mice injected with CIT or STZ and PBS or AMY. **(A)** Schematic representation of a sagittal mouse brain section from the Allen Brain Atlas. PS19 mice sections were stained with mAb AT8 (left panel) or mAb MC1 (right panel). **(B)** AT8 staining was quantified in the prefrontal cortex (PFC), hippocampus (H), and visual cortex (V. Cx). **(B, E)** STZ/AMY mice presented a larger AT8 and MC1^+^ stained area in the visual cortex in comparison with CIT/PBS and CIT/AMY (*P* < 0.05; *n* = 6 for all groups). **(C, D)** No differences were detected in the PFC (C), hippocampus (D), and other areas, such as the entorhinal cortex and amygdala (data not shown).

PS19 mice develop widespread tau pathology in the neocortex, amygdala, hippocampus, brain stem, and spinal cord.^46^ We assessed tau pathology in 6-month-old animals by immunostaining using tau antibodies that react with p-tau Ser202/Thr205 (mAb AT8) and pathological tau conformation (mAb MC1) (Fig. 4B). As expected, both antibodies revealed the broad distribution of tau pathology in sagittal brain sections. AT8-positive staining was quantified in several brain regions, such as the prefrontal cortex (Fig. 4C), hippocampus (Fig. 4D), and visual cortex (Fig. 4E). We observed that diabetes increased levels of tau pathology in the visual cortex as visualized using both AT8 (two-way ANOVA: diabetes factor, F_(1, 20)_ = 12.00, *P* *<* 0.01) (Fig. 4B–E). Additionally, Šídák's test showed that only STZ/AMY differed from the other groups. STZ/AMY exhibited higher AT8 positive staining than CIT/PBS (*P =* 0.05) and CIT/AMY (*P* *<* 0.05). Thus, STZ and amylin synergistically worsened tau pathology in certain brain regions of PS19 mice. Since the p-tau changes were detected only in the visual cortex, we focused on exploring the potential pathways underpinning the tau pathology changes in this region.

**Figure 5:**
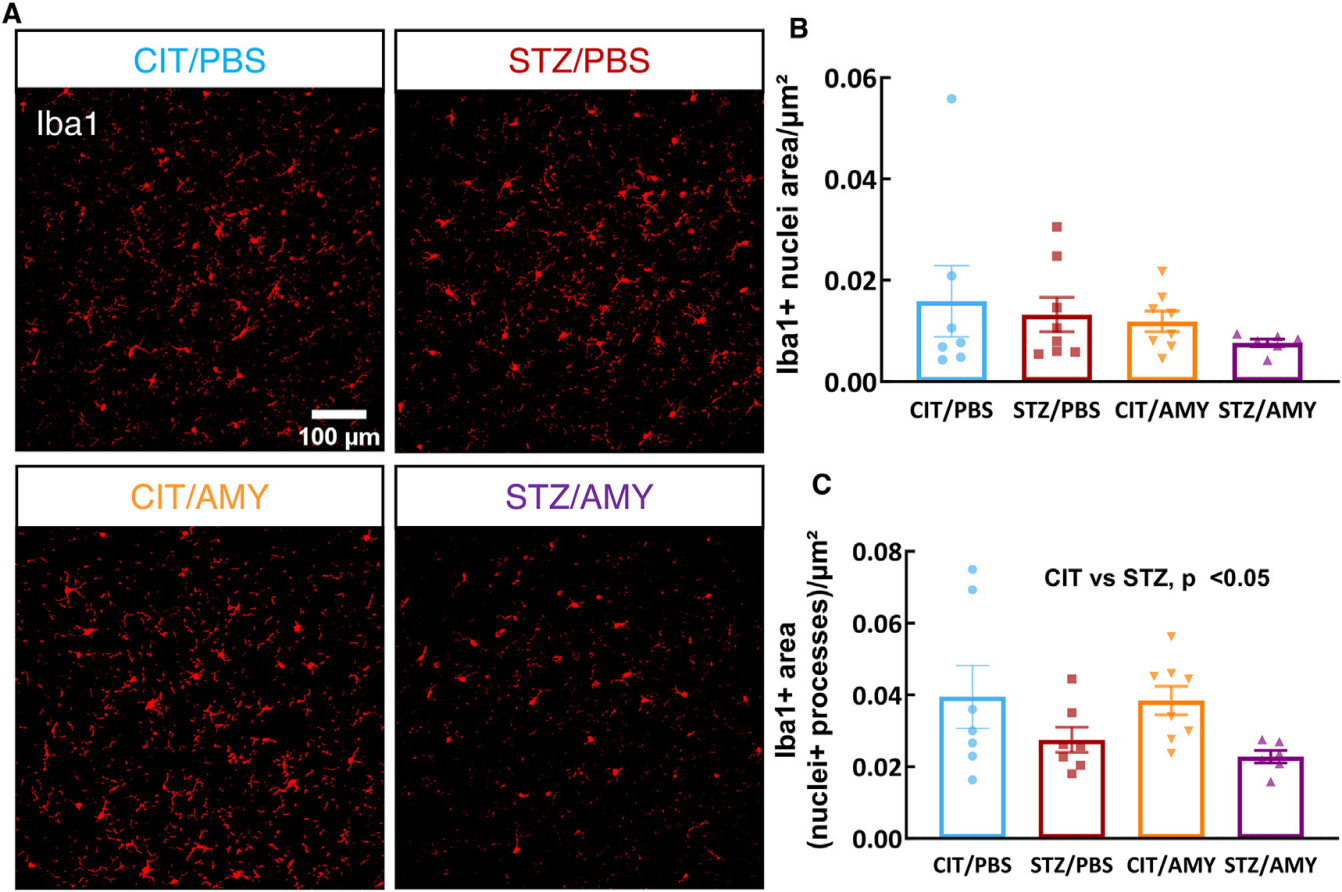
Immunostaining for IBA1 in the visual cortex of PS19 mice injected with CIT or STZ and PBS or AMY. **(A, B)** No differences were observed in the number of microglial cells detected in the visual cortex. **(C)** The quantification of the IBA1 staining in multiple fields of the visual cortex revealed a paucity of microglial processes in STZ-injected mice in comparison with the other groups (*P* < 0.05 for diabetes factor in Two-way ANOVA) (CIT/PBS, *n* = 7; STZ/PBS, *n* = 6; CIT/AMY, *n* = 8; CIT/AMY, *n* = 6).

**Figure S1:**
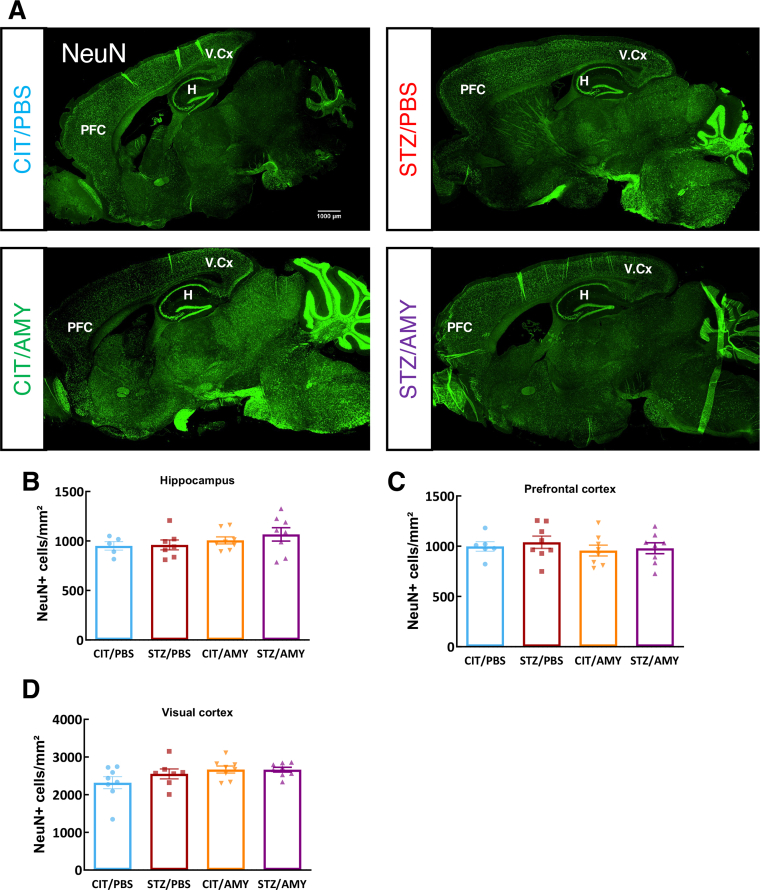
Immunostaining for NeuN in the brain of PS19 mice injected with CIT or STZ and PBS or AMY. **(A)** Representative images of NeuN staining. **(B**–**D)** The staining was quantified in the hippocampus (H) (B), prefrontal cortex (PFC) (C), and visual cortex (V. Cx) (D). No differences in the number of NeuN^+^ cells were detected (*n* = 5–8 animals per group).

**Figure S2:**
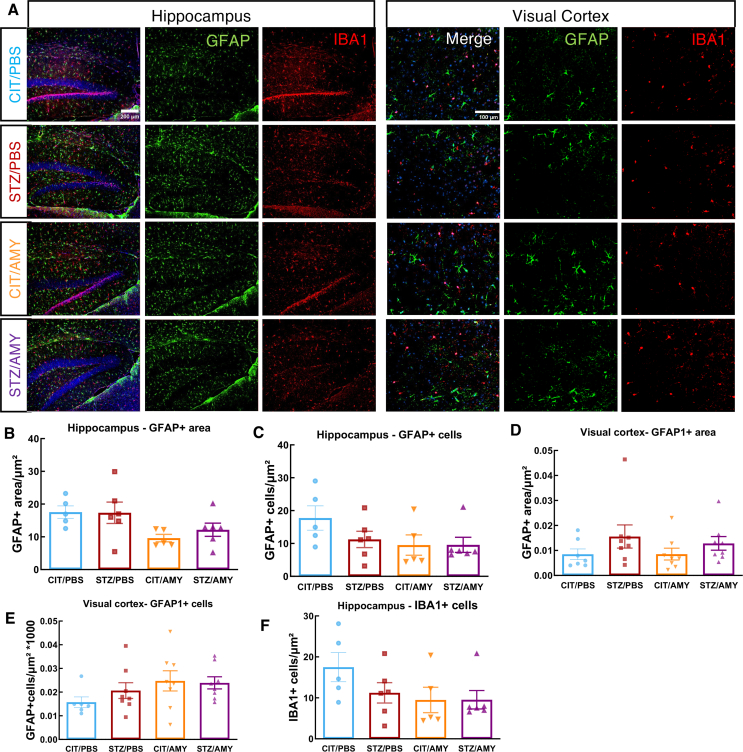
Immunostaining for GFAP and IBA1 in the brain of PS19 mice injected with CIT or STZ and PBS or AMY. **(A)** Representative images of GFAP and IBA1 staining in the hippocampus and visual cortex. **(B**–**E)** The quantification of GFAP^+^ area or number of cells in the hippocampus (H) (B, C) and visual cortex (V. Cx) (D, E) did not reveal any difference. **(F)** The number of IBA1^+^ cells was also similar between the groups (*n* = 5 or 6 animals per group).

Concomitant with tau pathogenesis and progression, PS19 mice exhibit profound neuronal loss and neuroinflammation.^46^ There was no difference in neuronal staining between the groups, as visualized by NeuN immunostaining (Fig. S1). We then assessed the extent of neuroinflammation in the visual cortex by immunostaining using cellular markers of astrocytes (glial fibrillary acidic protein, GFAP) and microglia (ionized calcium-binding adaptor molecule 1, Iba1). There was no significant difference in the density of Iba1^+^ microglia (Fig. 5A, B) or GFAP^+^ astrocytes in the visual cortex and the hippocampus (Fig. S2). However, microglial cells from STZ-injected mice displayed a smaller IBA1^+^-stained area (*i.e.*, fewer processes) in the visual cortex (two-way ANOVA: diabetes factor, F_(1, 24)_ = 6.726, *P* *<* 0.05) (Fig. 5C), suggesting that subtle differences in microglia may be related to increased p-tau burden in this brain region.

### Amylin synergizes with STZ, inducing lysosomal changes in the visual cortex of PS19 mice

**Figure 6:**
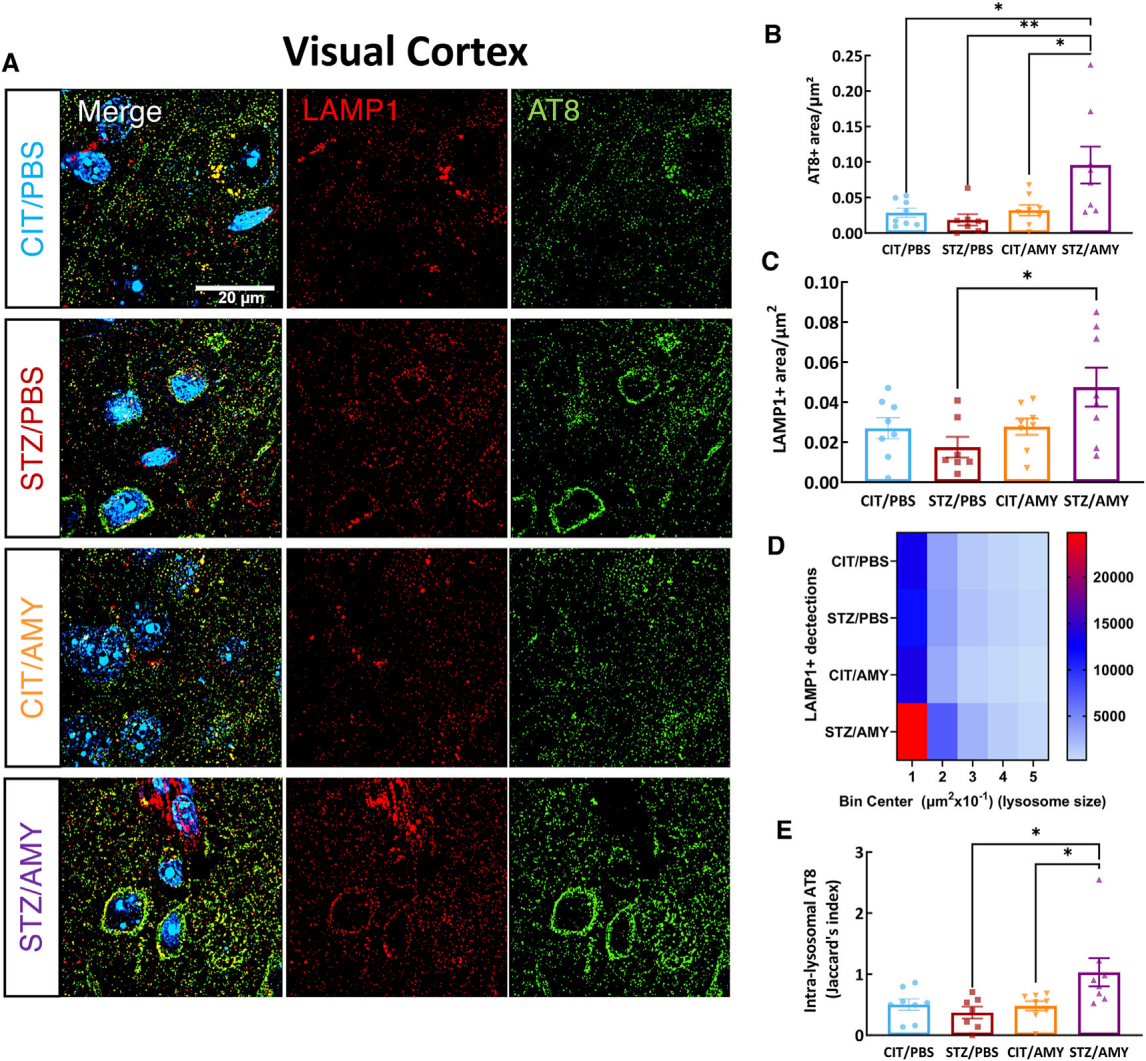
Immunostaining for LAMP1 and AT8 in the visual cortex of PS19 mice injected with CIT or STZ and PBS or AMY. **(A)** Representative images of AT8 (green), LAMP1 (red), and colocalization of AT8 and LAMP1 (merge) staining. Hoechst (blue) was utilized to stain the nuclei. **(B)** Quantification using SoRa imaging at 45 nm resolution (60 × objective and 4 × magnification) shows that STZ/AMY (*n* = 8) has a larger AT8^+^ area in the visual cortex in comparison with CIT/PBS (*P* < 0.06, *n* = 8), STZ/PBS (*P* < 0.01, *n* = 7), and CIT/AMY (*P* < 0.05, *n* = 8). **(C)** The quantification of the LAMP1^+^ area in multiple fields of the visual cortex revealed that STZ/AMY has a larger percentage of stained area in comparison with STZ/PBS (*P* < 0.05). **(D)** The heatmap illustrates that STZ/AMY has more LAMP1^+^ organelles than the other groups. **(E)** Jaccard's index (intersection divided by the union) shows that STZ/AMY has a higher co-localization of AT8 and LAMP1 in comparison with the other groups (*P* ≤ 0.05 for all comparisons).

Amylin is known to modulate lysosomal activity in the pancreas,^29^ and failures in autophagy are strongly correlated with tau pathology.^26^^,^^31^^,^^32^^,^^40^ Therefore, we investigated whether the worsening of tau pathology induced by amylin under diabetes conditions could be related to the modulation of lysosomal pathways in the visual cortex. We performed immunostaining using the lysosomal marker lysosomal-associated membrane protein 1 (LAMP1) along with the p-tau antibody AT8 and acquired high-resolution images (60X objective and SoRa 4x magnification) (Fig. 6A). AT8^+^ p-tau and LAMP1^+^ lysosomal staining were quantified across multiple fields of the visual cortex in each brain section, revealing significant differences between the groups. We observed an increase in p-tau levels (two-way ANOVA: amylin factor, F_(1, 27)_ = 7.377, *P* *<* 0.05; diabetes factor, F_(1, 27)_ = 3.242, *P =* 0.08; interaction, F_(1, 27)_ = 6.148, *P* *<* 0.05) in the visual cortex of AMY/STZ compared with all groups (Šídák's test: CIT/PBS, *P <* 0.05; STZ/PBS, *P* *<* 0.01; CIT/AMY, *P* *<* 0.05) (Fig. 6B). Additionally, the LAMP1^+^ area showed significant differences among the groups (F_(3, 27)_ = 3.681, *P* *<* 0.05). Post-hoc analysis indicated that the LAMP1^+^ area was larger in the visual cortex of STZ/AMY mice compared with STZ/PBS (two-way ANOVA: amylin factor, F_(1, 27)_ = 5.517, *P* *<* 0.05; interaction, F_(1, 27)_ = 4.983, *P* *<* 0.05; Šídák's test: STZ/PBS *vs*. STZ/AMY, *P* < 0.05) (Fig. 6C).

**Figure 7:**
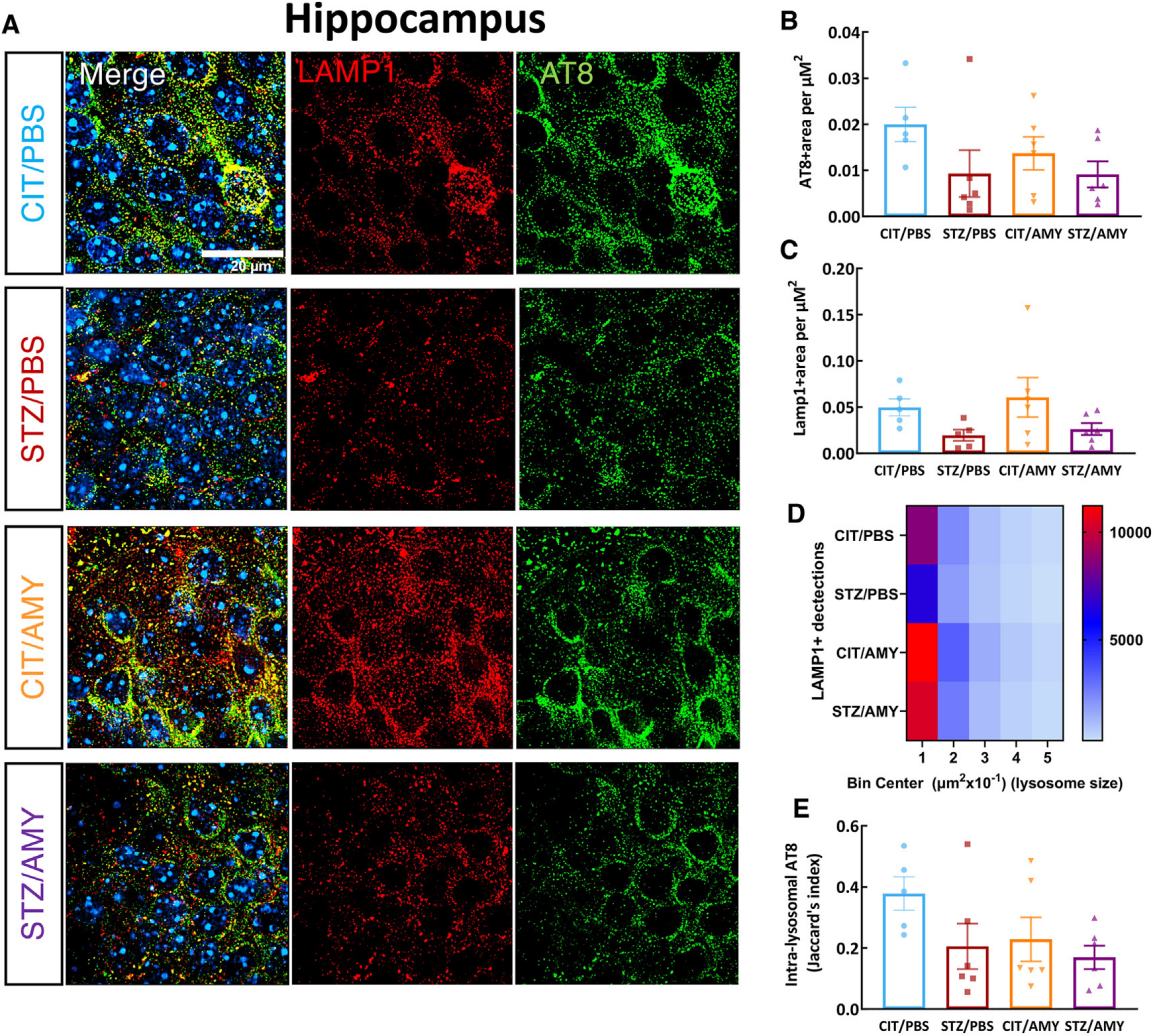
Immunostaining for LAMP1 and AT8 in the hippocampus of PS19 mice injected with CIT or STZ and PBS or AMY. **(A)** Representative images of AT8 (green), LAMP1 (red), and colocalization of AT8 and LAMP1 (merge) staining. Hoechst (blue) was utilized to stain the nuclei. **(B, C)** Quantification using SoRa imaging at 45 nm resolution (60 × objective and 4 × magnification) shows that the groups do not differ in AT8^+^ (B) and LAMP1^+^ (C) area in the hippocampus (CIT/PBS, *n* = 5; STZ/PBS, *n* = 6; CIT/AMY, *n* = 6; CIT/AMY, *n* = 6). **(D)** The heatmap illustrates that the CIT/AMY and STZ/AMY groups have a higher number and that the STZ/PBS group has a lower number of LAMP1^+^ organelles than the CIT/PBS group. **(E)** Jaccard's index (intersection divided by the union) shows that there is no difference in the co-localization of AT8 and LAMP1 between the groups (*P* ≤ 0.05 for all comparisons).

We then conducted a histogram analysis of the lysosomal size distribution. The heatmap illustrates the total number of LAMP1^+^ lysosomes detected in 4 images of randomly selected fields in the visual cortex of each animal, categorized into bins based on lysosome sizes. As shown in the heatmap, the numbers of LAMP1^+^ lysosomes across all sizes were greater in the STZ/AMY group (Fig. 6D; number of binned values: CIT/PBS = 19490; STZ/PBS = 18955; CIT/AMY = 18554; STZ/AMY = 36723). The Jaccard's index (intersection divided by the union) was calculated to assess the co-localization of LAMP1^+^ and AT8^+^ staining (two-way ANOVA: amylin factor, F_(1, 27)_ = 5.068, *P* *<* 0.05; interaction, F_(1, 27)_ = 5.806, *P* *<* 0.05). The Šídák's test indicated that the STZ/AMY group exhibited greater co-localization in comparison to CIT/PBS (*P =* 0.06), STZ/PBS (*P* *<* 0.05), and CIT/AMY (*P* *<* 0.05), suggesting that this group has a higher amount of intra-lysosomal p-tau in the visual cortex (Fig. 6E). AT8 and LAMP1 co-staining in the hippocampus showed no significant differences in AT8 levels among the groups (Fig. 7B). Although STZ-injected mice displayed a decrease in overall LAMP1 staining (two-way ANOVA: diabetes factor, F_(1, 18)_ = 5.912, *P* *<* 0.05) (Fig. 7C), post-hoc tests did not reveal significant inter-group differences, suggesting only subtle lysosomal changes in the hippocampus at this age. The intra-lysosomal levels of AT8 also did not differ between the groups (Fig. 7E). Interestingly, the number of small-sized LAMP1^+^ detections was higher in CIT/AMY (11,226) and STZ/AMY (10,393) but lower in STZ/PBS (6518) compared with CIT/PBS (8633) (Fig. 7D).

**Figure 8:**
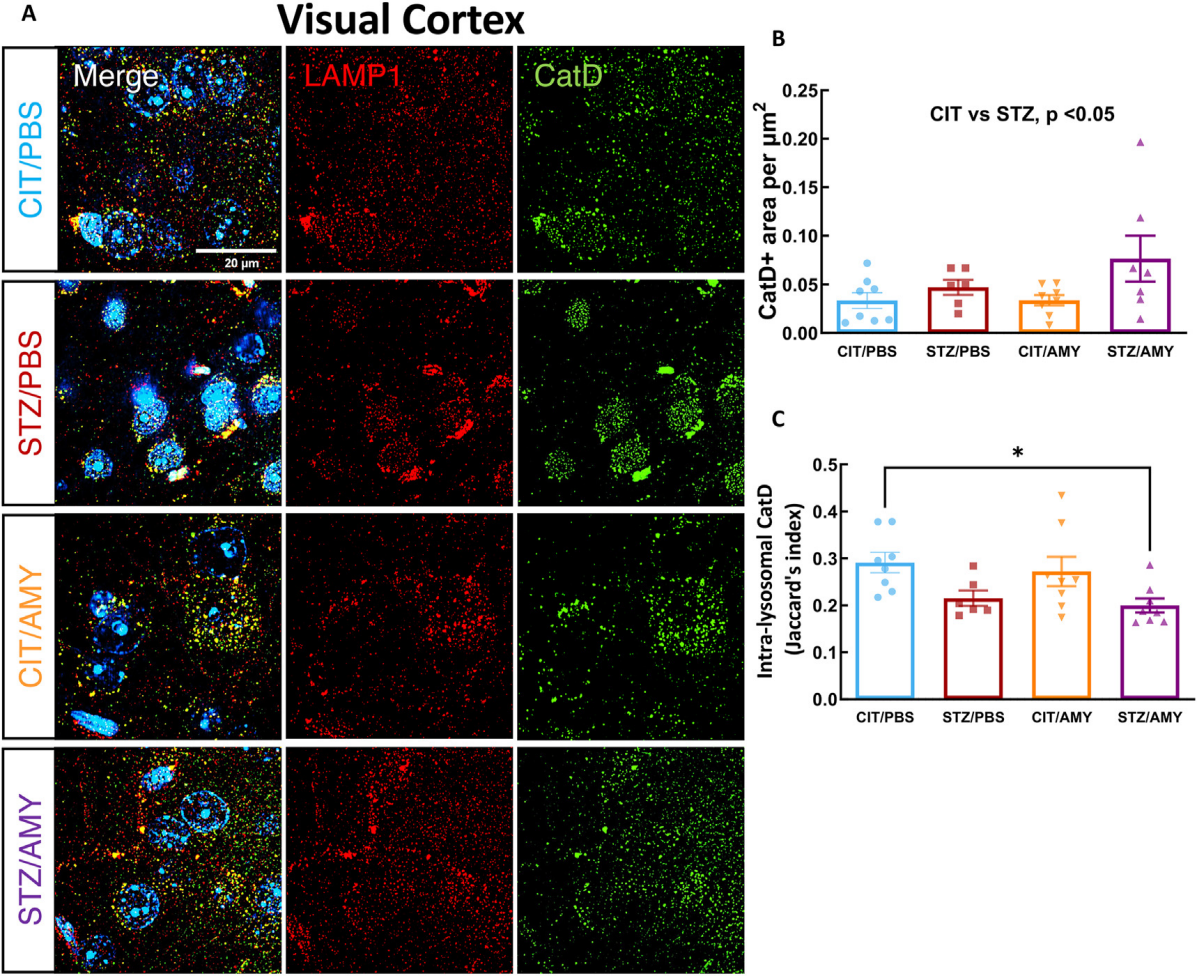
Immunostaining for LAMP1 and CatD in the visual cortex of PS19 mice injected with CIT or STZ and PBS or AMY. **(A)** Representative images of CatD (green), LAMP1 (red), and colocalization of AT8 and LAMP1 (merge) staining. Hoechst (blue) was utilized to stain the nuclei. **(B)** Quantification using SoRa imaging at 45 nm resolution (60 × objective and 4 × magnification) shows that CatD staining is not statistically different between the groups. **(C)** Jaccard's index (intersection divided by the union) shows that STZ/AMY has a lower co-localization of CatD and LAMP1 in comparison with the CIT/PBS (*P* < 0.05; CIT/PBS, *n* = 8; STZ/PBS, *n* = 6; CIT/AMY, *n* = 8; STZ/AMY, *n* = 8).

**Figure 9:**
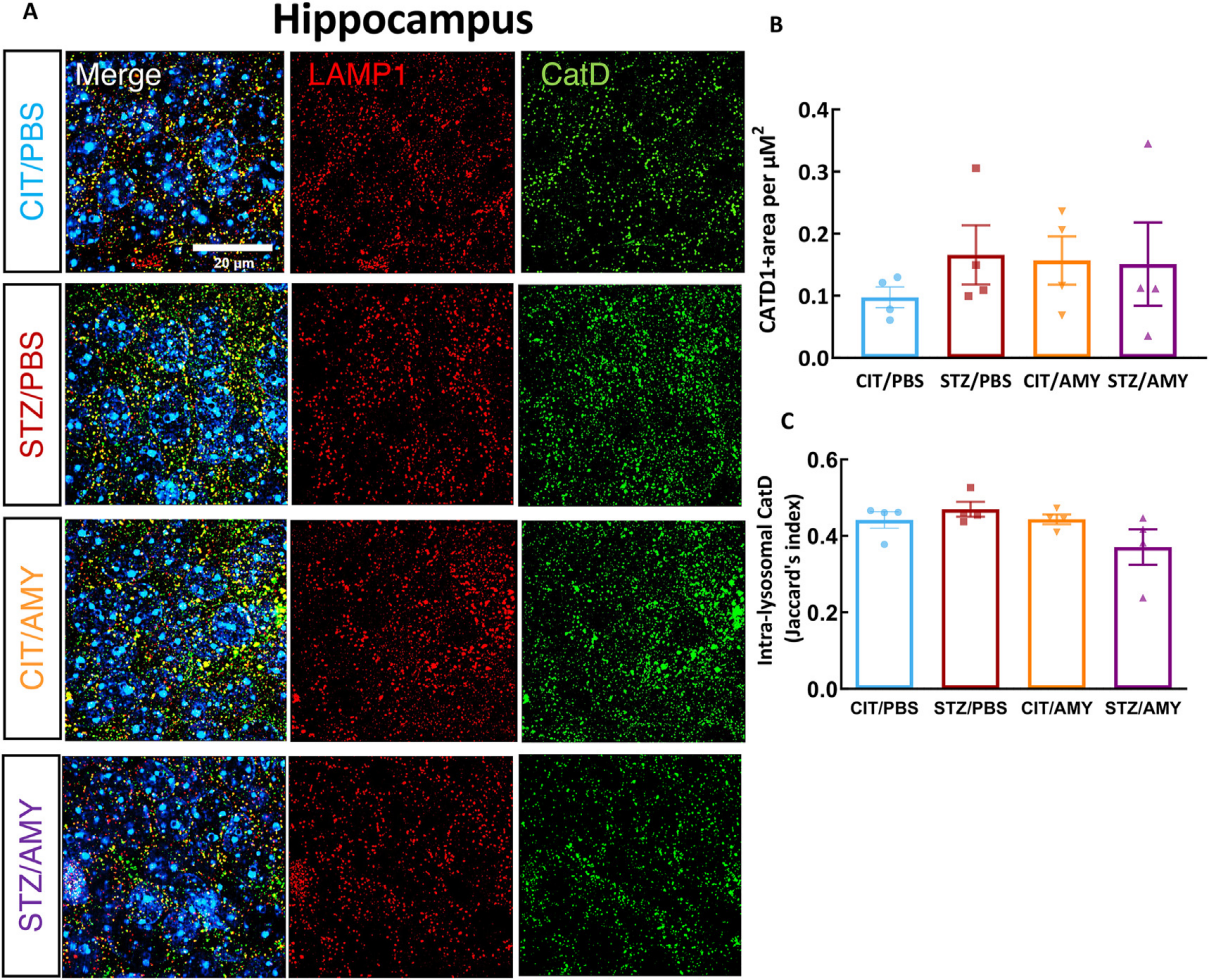
Immunostaining for LAMP1 and CatD in the hippocampus of PS19 mice injected with CIT or STZ and PBS or AMY. **(A)** Representative images of CatD (green), LAMP1 (red), and colocalization of AT8 and LAMP1 (merge) staining. Hoechst (blue) was utilized to stain the nuclei. **(B**–**D)** Quantification using SoRa imaging at 45 nm resolution (60 × objective and 4 × magnification) shows that CatD staining (C) and Jaccard's index (intersection divided by the union) (D) are not statistically different between the groups (CIT/PBS, *n* = 4; STZ/PBS, *n* = 4; CIT/AMY, *n* = 4; CIT/AMY, *n* = 4).

Next, we conducted an immunostaining analysis of the lysosomal protease CatD to investigate whether the accumulation of intra-lysosomal p-tau might result from lysosomal dysfunction. LAMP1^+^ and CatD^+^ immunostaining and co-localization were analyzed as above (Fig. 8A). The analyses indicated that the CatD^+^ area was increased in the visual cortex of STZ-treated mice (two-way ANOVA: diabetes factor, F_(1, 25)_ = 4.708, *P* *<* 0.05). However, the amylin factor and the interaction between amylin and diabetes were not significant, and post-hoc tests did not reveal significant inter-group differences (Fig. 8B). On the other hand, the intra-lysosomal presence of CatD was significantly lower (two-way ANOVA: diabetes factor, F_(1, 26)_ = 10.32, *P* < 0.05) in STZ/AMY compared with CIT/PBS (Šídák's test: *P* *<* 0.05) (Fig. 8C). These findings suggest that amylin could decrease the lysosomal functionality in the visual cortex of diabetic mice. In contrast, the immunostaining with LAMP1 and CatD in the hippocampus did not show differences between the groups regarding total CatD levels or intra-lysosomal CatD levels (Fig. 9). These results indicate that the overall reduction of lysosomes induced by STZ and the increase in the number of small lysosomes in the hippocampus promoted by amylin did not lead to loss of functionality in PS19 mice at the analyzed age, considering that p-tau levels were not elevated, CatD levels remained unaffected, and hippocampus-related behaviors were also not significantly impacted.

### STZ causes the loss of pancreatic islet cells and synergizes with amylin to elevate the levels of p-tau in the pancreas of PS19 mice

**Figure S3:**
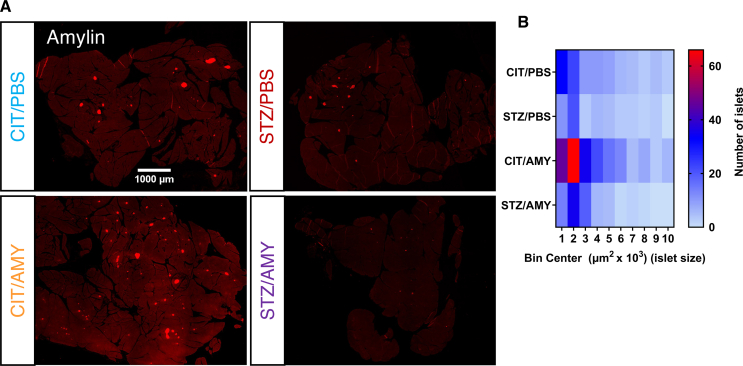
Immunostaining for amylin in the pancreas of PS19 mice injected with CIT or STZ and PBS or AMY. **(A)** Representative images of amylin staining in the pancreas of PS19 mice. **(B)** The quantification shows that CIT/AMY mice have approximately twice the number of pancreatic islets compared with the other groups (*n* = 6 animals per group).

Next, we conducted immunostaining analyses of the pancreas to investigate peripheral pathological changes associated with the STZ-diabetes model and to explore the possible extent of tau pathology changes induced by amylin outside the central nervous system. First, we stained the pancreas with amylin antibody and counted the number of pancreatic islets to assess the extent of pancreatic islet cell death due to STZ's toxic effect (Fig. S3A). The number of islets was reduced in the STZ/PBS and STZ/AMY groups, as expected in the STZ-diabetes model (Fig. S3B). Interestingly, the CIT/AMY group presented a larger number of pancreatic islets of all sizes (Fig. S3B) (number of pancreatic islets: CIT/PBS = 144; STZ/PBS = 57; CIT/AMY = 265; STZ/AMY = 93), suggesting that amylin *per se* can be protective against the loss of pancreatic islet cells. The number of islets was calculated by summing all the islets detected in images obtained from whole section scans (4 × objective, 5× 5 fields) of 6 different animals per group (2 sections per animal and 12 per group). For instance, considering the bin size of 2 μm^2^ × 10^3^ illustrated in the heatmap, the number of islets is as follows: CIT/PBS = 23, STZ/PBS = 21, CIT/AMY = 66, and STZ/AMY = 35; and for the bin of 10 μm^2^ × 10^3^, the numbers were CIT/PBS = 3, STZ/PBS = 0, CIT/AMY = 6, and STZ/AMY = 0.

**Figure 10:**
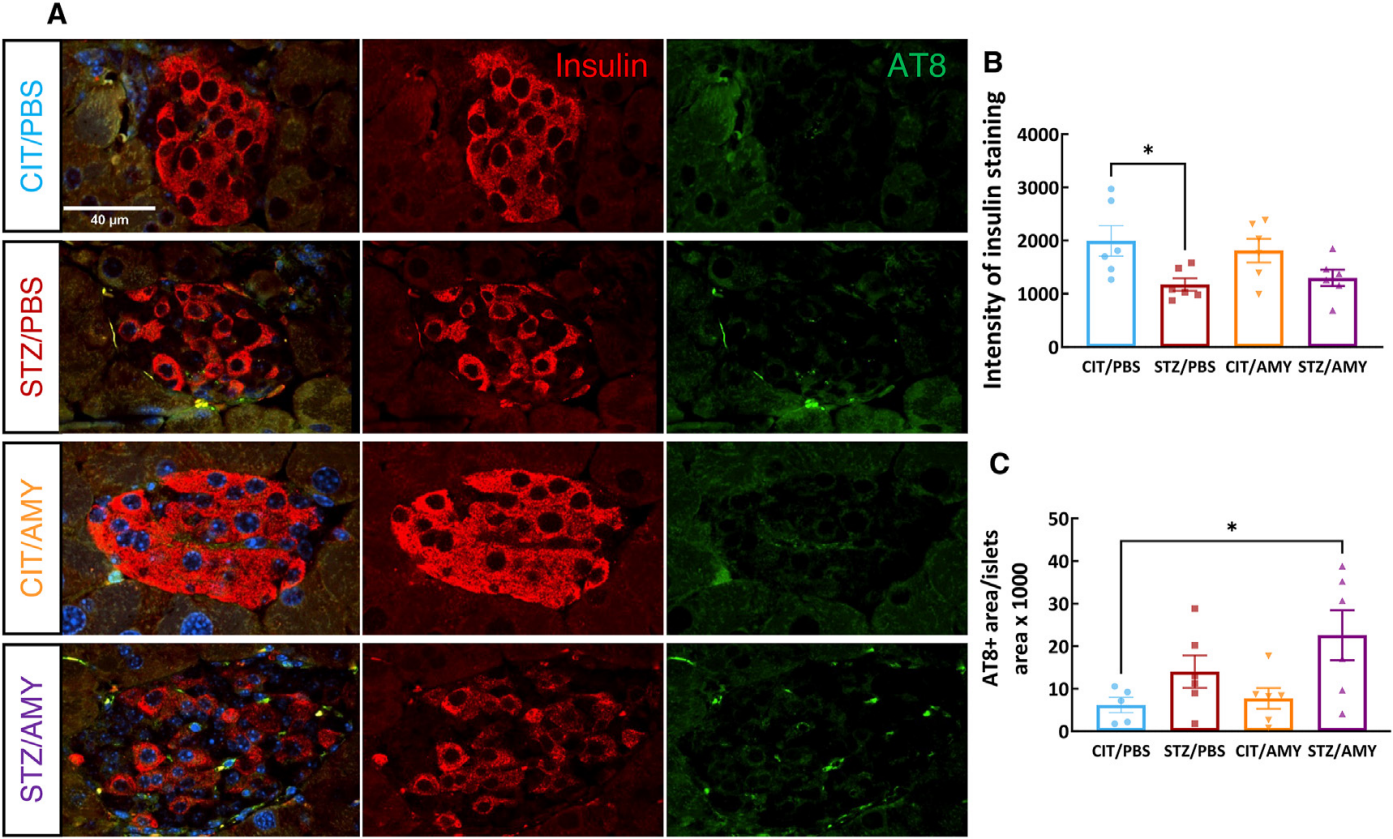
Immunostaining for insulin and AT8 in the pancreatic islets of PS19 mice injected with CIT or STZ and PBS or AMY. **(A)** Representative images of AT8 (green), insulin (red), and co-staining of AT8 and LAMP1 (merge) staining. Hoechst (blue) was utilized to stain nuclei. **(B)** Quantification of insulin staining shows that STZ decreases the intensity of insulin staining in the pancreatic islet cells (*P* = 0.12, STZ/AMY *vs*. CIT/PBS; *P* < 0.05, STZ/PBS *vs*. CIT/PBS. **(D)** Quantification of AT8 staining shows that STZ/AMY has a larger AT8^+^ area than CIT/PBS (*P* < 0.05). *n* = 6 for all the groups.

We then performed co-immunostaining using insulin and AT8 antibodies to determine whether amylin would synergize with STZ to influence tau pathology in the pancreatic islets, which were positively stained with insulin (Fig. 10A). The intensity of insulin staining was reduced in STZ-injected mice (two-way ANOVA: diabetes factor, F_(1, 20)_ = 10.57, *P* *<* 0.01). Šídák's test revealed that STZ/PBS and STZ/AMY were slightly lower compared with CIT/PBS (*P* = 0.051 and *P* = 0.125, respectively), representing another feature of the diabetic phenotype (Fig. 10B). Interestingly, the AT8^+^ stained area in the pancreatic islets was larger (two-way ANOVA: diabetes factor, F_(1, 19)_ = 8.165, *P* *<* 0.01) in STZ/AMY in comparison to CIT/PBS (Šídák's test: *P* < 0.05). The stained area in the STZ/AMY group also tended to be greater than that of the CIT/AMY group (Šídák's test: *P* < 0.06). This study provides the first evidence that amylin exacerbates tau pathology not only in the brain but also in the pancreas of STZ-induced diabetic PS19 mice, as shown in Figure 10C.

**Figure 11:**
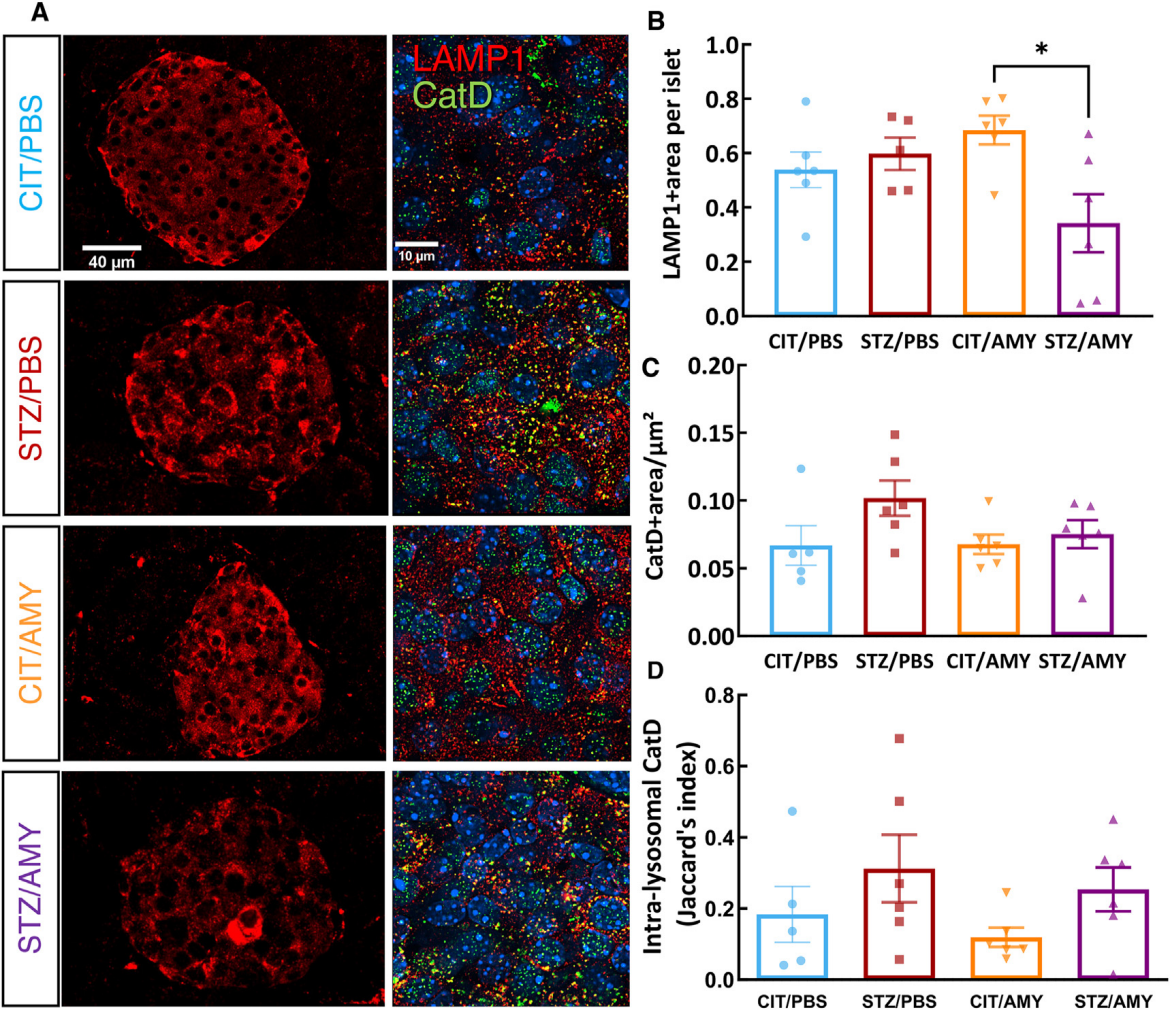
Immunostaining for insulin, LAMP1, and CatD in the pancreatic islets of PS19 mice injected with CIT or STZ and PBS or AMY. **(A)** Representative images of LAMP1 (red) staining (20×) and co-staining of LAMP1 and CatD (SoRa imaging 60× objective with 4× magnification) inside the islets. The nuclei were stained with Hoechst (blue). **(B)** Quantification of LAMP1 staining shows that STZ/AMY has a smaller LAMP1^+^ area in the pancreas than CIT/AMY (*P* < 0.05). **(C)** CatD staining was not different between the groups.

As described above, amylin administration in PS19 mice impacted the lysosomal functionality in the visual cortex concomitant with the worsening of tau pathology (Figure 4, Figure 6, Figure 8). Thus, we asked whether the observed increase in p-tau in the pancreas may also relate to lysosome alterations. Co-immunostaining with antibodies against the lysosomal markers LAMP1 and CatD (Fig. 11A, B) revealed that the STZ/AMY group exhibited a lower proportion of LAMP1^+^ lysosomes within pancreatic islets compared with the CIT/AMY group (two-way ANOVA: interaction F_(1, 19)_ = 7.006, *P* *<* 0.05; Šídák's test: *P* *<* 0.05), suggesting that amylin synergized with STZ, to reduce lysosomal density in the pancreatic islet cells. However, the CatD^+^ area (Fig. 11C) and the intra-lysosomal presence of CatD (Fig. 11D) in the pancreatic islets remained unchanged between the groups.

## Discussion

Our study found that peripheral amylin administration exacerbated tauopathy by impairing lysosomal mechanisms in a hybrid model of diabetes and tau pathology based on STZ intraperitoneal injections in PS19 mice. STZ administration mimicked typical features of diabetes such as weight loss^53^ and elevated blood glucose levels,^54^^,^^55^ which exceeded 200 mg/dL in 100 % of the STZ-injected mice. On the other hand, amylin treatment did not have a long-term effect on blood glucose levels, consistent with previous evidence that the amylin-decreasing effect on blood glucose and food intake lasts for 2 h or less after the peptide enters the bloodstream.^56^^,^^57^ Nevertheless, mice administered with STZ and amylin were observed to have worsened motor coordination and elevated p-tau levels in the visual cortex and pancreas at 6 months, concurrent with signs of lysosomal decompartmentalization and dysfunction. Based on our observations, we suggest that amylin synergizes with STZ to compromise lysosomal integrity and tau clearance, thereby exacerbating tau pathology in the visual cortex of mice at 6 months of age. As tau pathology is known to propagate through anatomically connected brain regions, we will explore in future studies whether STZ/amylin-mediated enhancement of pathology becomes evident in other brain regions in older PS19 mice.

Previous studies have reported that the administration of STZ leads to a reduction in gastrocnemius twitch force, a decrease in the size of motor neurons,^58^ and atrophy of the hindlimb representation area in the cortex of rats.^59^ In agreement, the STZ-administered mice performed poorly in the ledge and hindlimb clasping tests, revealing deficits in hindlimb strength and control. These findings are consistent with previous evidence that the STZ animal models replicate the motor difficulties and loss of muscle strength observed in patients with diabetes.^58^, ^59^, ^60^ We also found that STZ increased the fear-conditioning freezing response. Although strong evidence suggests that diabetes patients are more prone to experience memory impairments,^61^, ^62^, ^63^ there is no consensus on STZ's effect on fear conditioning in rodents. While a single injection of STZ (65 mg/kg) did not impact contextual fear conditioning in rats,^64^ another study that used a different dose (50 mg/kg) in rats reported a behavioral response similar to our findings. The diabetic animals displayed a higher freezing percentage during the training, test, and re-exposure sessions in a different context, indicating a generalization of the fear memory.^65^ Our results, though unexpected, may reflect diabetes-related compensatory mechanisms at early disease stages, which could temporarily enhance fear-associated learning.

The reason why amylin did not affect cognitive performance is unclear, as there are conflicting reports from studies that utilized different treatment protocols and animal models. The administration of amylin or its analog, pramlintide (200 μg/kg), for 30 days did not alter behavioral responses in Tg-SwDI amyloid precursor protein (APP) transgenic mice but impacted amyloid deposition in the brain.^66^ Utilizing the same drug in a dementia model based on STZ intracerebroventricular injection in rats, Nassar and colleagues reported that a five-week pramlintide administration (200 μg/kg) improved learning and memory while decreasing levels of p-tau and β-amyloid in the hippocampus of STZ-injected rats.^67^ On the other hand, the intracerebral injection of pancreas homogenate from human amylin transgenic mice accelerated Aβ deposition and worsened memory in Tg2576 APP transgenic mice when compared with untreated Tg2576 controls or those that received the homogenates from STZ-diabetic mice.^68^ Our findings indicate that despite not affecting cognitive behavior, amylin significantly exacerbated tauopathy in the visual cortex of diabetic tau transgenic mice (STZ/AMY group), as observed by immunostaining with AT8 and MC1 antibodies selective for p-tau (Fig. 4).

**Figure S4:**
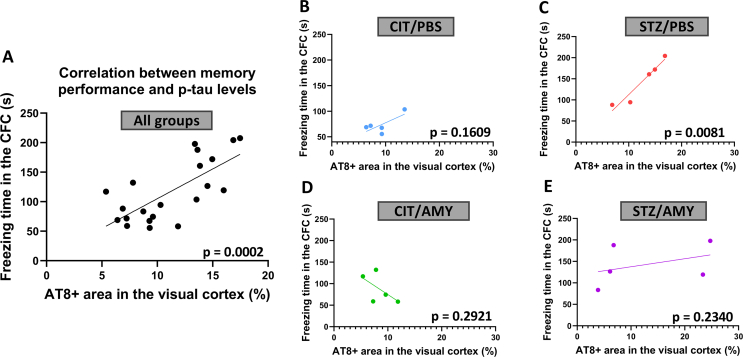
Simple linear regression analysis between p-tau levels in the visual cortex and the freezing in the contextual fear conditioning test of PS19 mice injected with CIT or STZ and PBS or AMY. **(A)** Correlation across all samples. **(B**–**E)** Correlation of values for the CIT/PBS (B), STZ/PBS (C), CIT/AMY (D), and STZ/AMY (E). The correlation is strongly positive (*P* < 0.0001) across all samples. When analyzed within the group, only the STZ/PBS group showed a significant correlation (*P* < 0.01), whereas CIT/PBS, CIT/AMY, and STZ/AMY failed to do so (*n* = 5 animals per group).

Interestingly, we observed a correlation between memory performance and p-tau levels in the visual cortex of PS19 mice (Fig. S4; *P* < 0.0001). While the STZ/PBS group displays a strong correlation (*P* < 0.01), this correlation is disrupted in the CIT/AMY and STZ/AMY mice (*P* > 0.05 for both groups), emphasizing the impact of amylin treatment on the relationship between tauopathy in the visual cortex and fear memory. The visual cortex is an area affected by both AD^69^ and diabetes^70^ and is associated with visual memory impairments.^71^ Furthermore, the visual cortex also has a strong binding activity of amylin's pharmacological targets, the calcitonin receptors.^72^ It has been reported that amylin structurally interacts with tau and contributes to its aggregation.^21^, ^22^, ^23^, ^24^ The injection of co-aggregates of tau and amylin into the hippocampus of PS19 mice induced more severe tauopathy along with increased synaptic and cognitive impairments compared with the effects of tau injection alone.^21^ Moreover, the co-expression of human tau and amylin in mice led to heightened insulin resistance, glucose intolerance, and tau phosphorylation.^24^ While we observe a pathological impact of peripheral amylin on tauopathy in the visual cortex of 6-month-old mice, older animals might exhibit a more extensive exacerbation of tau pathology in other brain regions. Our goal was to determine whether the treatment group deviated from this expectation, exhibiting an earlier pathological burden induced by diabetes and amylin synergism. Nonetheless, longitudinal studies in diabetic tauopathy models are necessary to assess how these early changes may progress to other brain regions, influencing the severity of pathology and behavioral performance as the disease advances. Moreover, while the PS19 model captures specific aspects of diabetic tauopathy, future investigations using additional models are essential to validate these findings in other models of AD-associated tau pathophysiology.

Our findings in the PS19 model are consistent with published reports showing that the pathology is more severe in males than in females. The systematic study conducted by Sun et al details that PS19 mice exhibit stronger phenotypes regarding weight loss, survival rate, composite phenotype assessment, tau phosphorylation, and neuroinflammation, representing a more sensitive platform for investigating tauopathy.^48^ Given the well-documented sexual differences in behavior, neuropathology, and plasma protein profiles in PS19 transgenic AD mice,^48^ we designed our study to perform group comparisons within a single sex. However, the use of only males in our study remains an important limitation to consider in future research. While we did not observe substantial amylin-induced changes across many brain regions, it is possible that females, which show a milder tauopathy, may exhibit a more pronounced amylin effect. Future investigations should examine whether the same lysosomal pathways, specific brain regions, and pancreatic alterations identified in males are similarly impacted in females. Additionally, it is essential to broaden our findings in future investigations by implementing a standardization of STZ dosing appropriate for both sexes to elucidate the broader implications of amylin's effects and enhance the translational significance of our findings.

**Figure S5:**
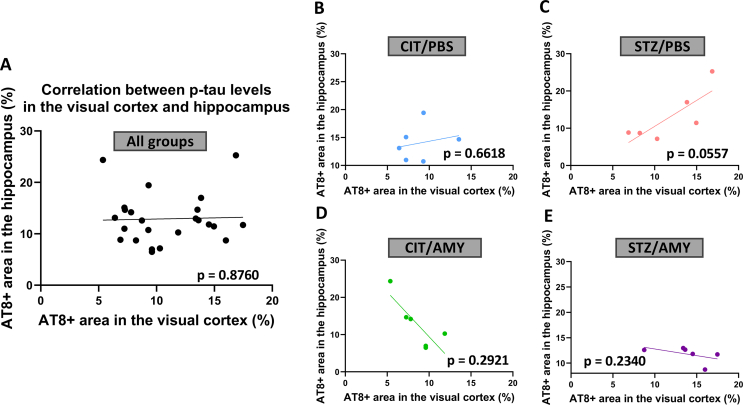
Simple linear regression analysis between the p-tau levels in the visual cortex and the hippocampus of PS19 mice injected with CIT or STZ and PBS or AMY. **(A)** Correlation across all samples. **(B**–**E)** Correlation of values for the CIT/PBS (B), STZ/PBS (C), CIT/AMY(D), and STZ/AMY (E). The correlation tends to be positive in STZ/PBS (*P* = 0.055). CIT/PBS, CIT/AMY, and STZ/AMY did not present a significant correlation (*n* = 5 animals per group).

Neither STZ nor amylin administration led to significant neuronal death or changes in the number of astrocytic and microglial cells. However, microglia in the visual cortex of STZ/AMY mice exhibited fewer microglial processes with shortened ramification (Fig. 5). It is known that resting microglia possess longer ramified processes, which shorten as the cells undergo morphological and functional transitions to an activated state in response to injury and inflammation, including accrual of tau pathology.^73^ Given that the disruption of the autophagy-lysosomal pathway in AD contributes to tau accumulation,^74^ we further examined the presence of p-tau within lysosomes in the visual cortex using high-resolution microscopy co-localization analysis of LAMP1 and p-tau immunostaining. Notably, STZ/AMY mice displayed a significant increase in lysosomes, particularly smaller-sized lysosomes, along with a substantially larger overall LAMP1^+^ stained area compared with the STZ/PBS group (Fig. 6D). Moreover, the intra-lysosomal p-tau levels were elevated in STZ/AMY mice, suggesting an increase in lysosomes in response to p-tau burden (Fig. 6E). Nevertheless, the decrease in intra-lysosomal localization of CatD, a lysosomal enzyme involved in tau, β-amyloid, and p-tau degradation, in STZ/AMY mice indicated possible leakage of this enzyme from damaged lysosomes into the cytosol (Fig. 8). Evidence suggests that the leakage of lysosomal hydrolytic enzymes such as CatD into the cytosol initiates a slow apoptotic process by fragmenting structures within cells that still possess intact plasma membranes.^75^ A similar slow apoptotic process may explain why STZ/AMY mice do not exhibit significant cell death in any brain region analyzed (Fig. S1), despite showing increased p-tau burden and dysfunctional lysosomes. Intriguingly, although the hippocampus demonstrated no differences in p-tau burden or LAMP1^+^ area across the groups (Fig. 7), there was a higher count of small-sized LAMP1^+^ detections in both the CIT/AMY and STZ/AMY groups, whereas the STZ/PBS group showed a lower count compared with the CIT/PBS group (Fig. 7D). Unlike the findings in the visual cortex, CatD levels were not significantly different in the hippocampus across the groups (Fig. 9). Collectively, our study suggests that amylin increases lysosome numbers in both the cortex and hippocampus of diabetic PS19 mice. However, lysosomal dysfunction in the cortex (reflected by reduced intra-lysosomal CatD) hinders tau clearance, contributing to tauopathy. In contrast, intact lysosomal function in the hippocampus likely prevents excess tau accumulation at this age, which may explain the absence of memory deficits in our model. The subtle correlation in p-tau levels between the visual cortex and hippocampus observed only in the STZ/PBS group (Fig. S5) further suggests that the amylin-induced increase in lysosome in the hippocampus, combined with stable intra-lysosomal CatD levels, may protect this region from early tau pathology exacerbation seen in the visual cortex at 6 months of age.

**Figure S6:**
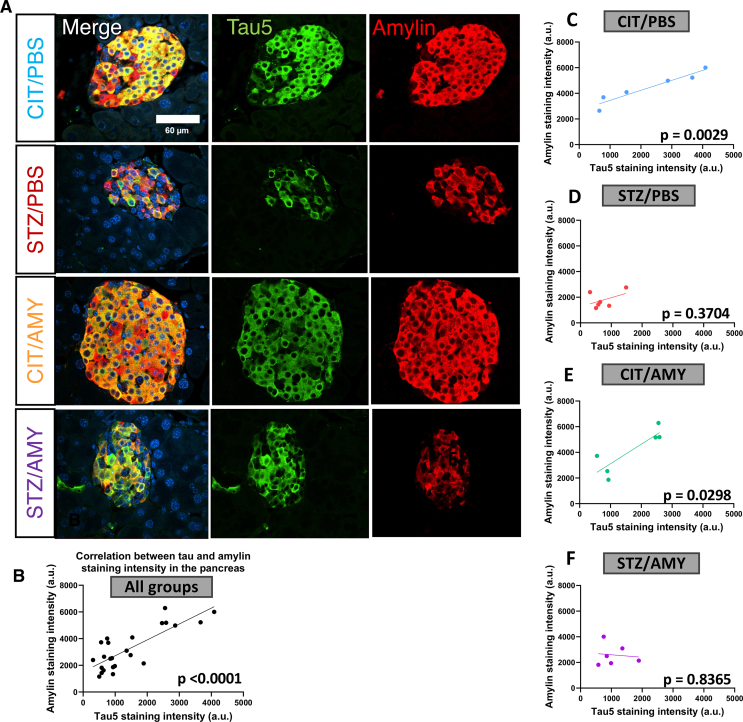
Simple linear regression analysis between the total tau levels and amylin in the pancreatic islets of PS19 mice injected with CIT or STZ and PBS or AMY. **(A)** The correlation across all samples. **(B**–**E)** Correlation of values for the CIT/PBS (B), STZ/PBS (C), CIT/AMY(D), and STZ/AMY (E). The correlation is strongly positive (*P* < 0.0001) across all samples. When analyzed within the group, CIT/PBS and CIT/AMY showed a significant correlation (*P* < 0.01), whereas STZ/PBS and STZ/AMY did not present a significant correlation (*n* = 6 animals per group).

Since amylin impairs lysosomal activity in the pancreas,^29^ we further investigated its possible effects on tau pathology and lysosomes in the pancreatic islets. Interestingly, CIT/AMY mice displayed a higher number of pancreatic islets compared with the other groups, indicating that amylin helped prevent the death of pancreatic β-cells in PS19 mice (Fig. S3). As expected, insulin staining intensity was lower in the STZ-treated mice due to the death and loss of functionality of pancreatic β-cells (Fig. 8). Moreover, STZ/AMY mice showed a higher percentage of AT8-positive area in the islets compared with all the other groups. This finding agrees with previous evidence that p-tau is also found in peripheral organs, including the pancreas of diabetic rats^76^ and humans.^77^ The immunostaining for total tau and amylin (Fig. S6) reveals a significant positive correlation between total tau and amylin staining in the pancreas (*P* < 0.0001), suggesting a shared relationship driven by amylin's propensity to aggregate and promote tau stabilization or aggregation. This correlation was strongest in CIT/PBS and CIT/AMY mice, where islet health was maintained. In STZ-treated mice, beta cell destruction decreased islet-derived amylin, weakening the tau-amylin relationship. Overall, our findings indicate that diabetes disrupts tau-amylin dynamics, whereas amylin treatment affects tau aggregation, lysosomal activity, and islet health in a context-dependent manner. Our data suggests that amylin may increase the risk of diabetic mice developing tau pathology not only in certain brain regions but also in the pancreas, reinforcing that diabetes and AD are intricately connected and mutually influencing each other. Amylin and STZ also synergized to inhibit lysosomal presence in the pancreas, possibly contributing to increased p-tau in pancreatic islet cells. Autophagy plays a crucial role in the homeostatic activity of pancreatic β-cells, and failures in autophagic mechanisms lead to pancreatic degeneration, impaired insulin secretion, and glucose intolerance, all hallmarks of diabetes.^36^ Muralidharan et al provided evidence of lysosomal dysfunction in the pancreatic β-cells of mice and patients with type 1 diabetes even before the onset of clinical hyperglycemia symptoms.^78^ In a study conducted with individuals at different stages of AD, tauopathy and β-amyloid pathology were observed only at mild and severe stages. However, an elevation in the plasma levels of CatD and other lysosomal markers was observed even in the mild cognitive impairment stage.^79^ In this context, identifying autophagy markers in diabetes and AD emerges as a promising biomarker that could aid in the diagnosis of both diseases and serve as a future therapeutic target for further exploration. Several autophagy modulators, including curcumin and rapamycin (mammalian target of rapamycin (mTOR) inhibitors) and resveratrol and metformin, (AMP-activated protein kinase activators), have been studied in AD animal models and shown to be safe in various phases of clinical trials, despite being initially developed for treating conditions other than AD.^36^ Translating findings from the PS19 mouse model of tauopathy to human AD presents several challenges due to species-specific differences in pathophysiology, metabolic processes, and disease progression. While PS19 mice develop tau pathology that resembles human AD, differences in regional distribution, disease progression, and the interplay between amylin and tau in diabetes may limit direct translation. To bridge this gap, comparative studies examining the similarities and differences between PS19 mice and human AD patients can help clarify the model's relevance. Genomic and proteomic analyses of the pancreas and brain in diabetic and AD patients would be valuable for pinpointing key molecular targets at the intersection of metabolic peripheral alterations and neurodegeneration. Additionally, preclinical studies utilizing human neuronal cell lines or induced pluripotent stem cell-derived neurons could help elucidate amylin's effects on tau pathology at a mechanistic level. Advanced imaging techniques such as positron emission tomography scans and longitudinal studies in both models can further assess disease progression and therapeutic relevance, ultimately enhancing translation to clinical applications.

**Figure 12:**
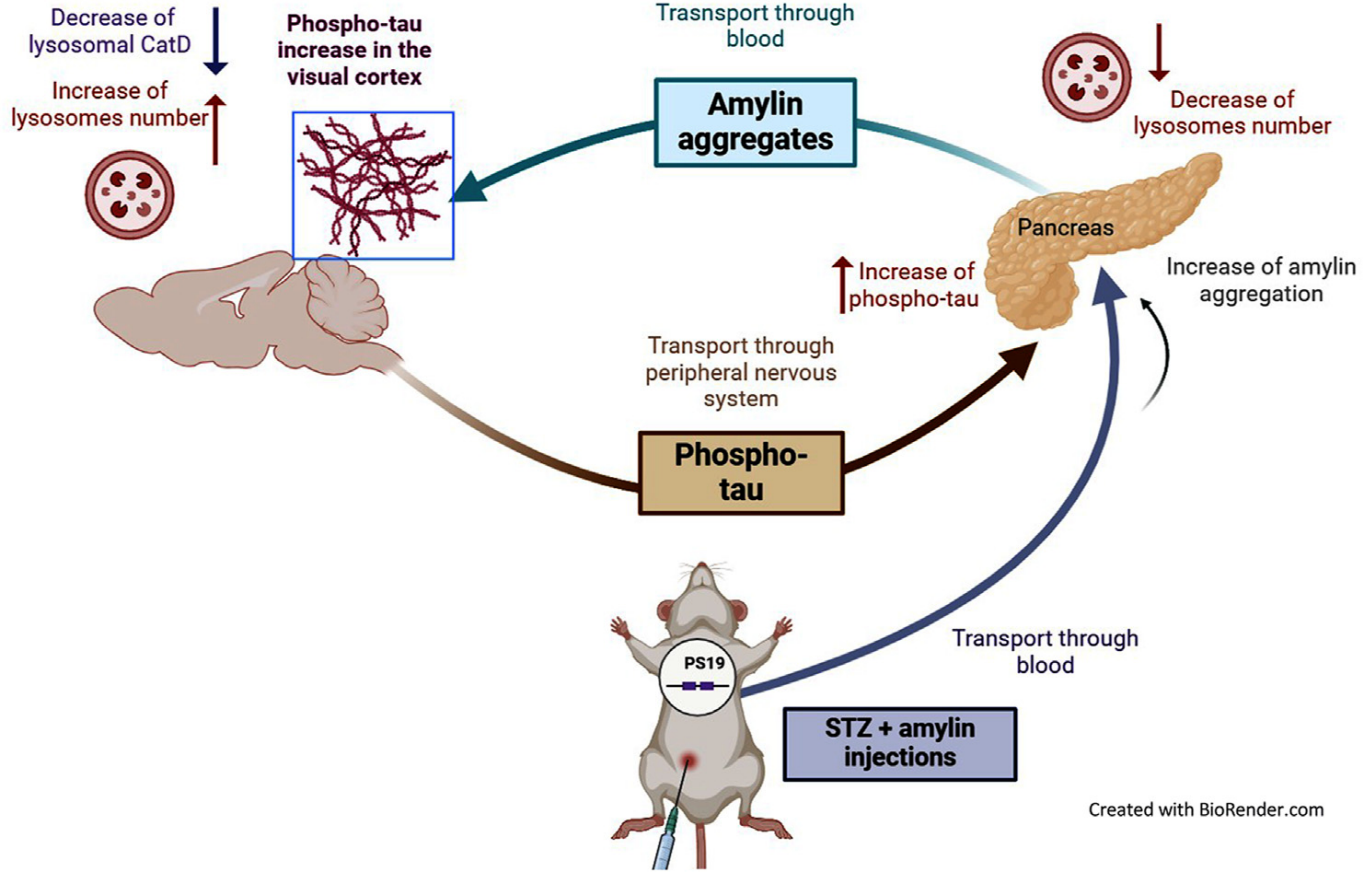
Graphical summary of the possible connection between amylin and tau pathology in the brain and pancreas of diabetic PS19 mice. The intraperitoneal administration of amylin aggregates caused the emergence of pathogenic p-tau concomitant with a decrease in lysosomes in the pancreas of diabetic PS19 mice. Although the plasmatic amylin remained unchanged in mice at 6 months of age, we speculate that a fraction of amylin aggregates may have reached the brain during the two-week injection period (4.5 months). Peripheral amylin aggregates, through a yet unknown mechanism, contributed to the exacerbation of p-tau in the visual cortex. The increase of p-tau in the visual cortex was accompanied by decreased lysosome functionality. However, the chronological order of these events still needs to be clarified. Further research is necessary to fully understand the spatial and temporal order and the mechanisms underlying the involvement of amylin in the aggregation of tau in both the central nervous system and the periphery.

In summary, we propose a mechanistic model that could explain the modulatory action of amylin on lysosomal activity in diabetic tau transgenic mice, exacerbating tau pathology in both the periphery and the nervous system (Fig. 12). During diabetes, amylin can reduce lysosomal activity in the pancreas, potentially promoting amylin aggregation and its transport to the brain. Consequently, amylin aggregates worsen tau pathology in the visual cortex by decreasing the presence of intra-lysosomal CatD. Additionally, we speculate that p-tau spreads from the brain to the peripheral neurons in the pancreas, which may contribute to worsening metabolic dysfunctions in the periphery. Further studies are needed to fully elucidate the molecular pathways through which amylin and tau are transferred between the periphery and the brain.

## CRediT authorship contribution statement

**Daniel Moreira-Silva:** Writing – review & editing, Writing – original draft, Visualization, Validation, Methodology, Investigation, Formal analysis, Data curation, Conceptualization. **Melike Yuksel:** Investigation, Formal analysis, Data curation. **Moorthi Ponnusamy:** Investigation, Formal analysis. **Mitchell T. Hansen:** Investigation, Formal analysis. **Joseph D. McMillan:** Investigation, Formal analysis. **Sneha Geethakrishnan:** Formal analysis. **Shuai Wang:** Formal analysis. **Lisa A. Collier:** Project administration. **Gopal Thinakaran:** Writing – review & editing, Supervision, Resources, Project administration, Funding acquisition, Formal analysis, Conceptualization.

## Ethics declaration

All experimental procedures related to animal care and experimental manipulation were conducted in accordance with the policies of the Institutional Animal Care and Use Committee at the University of South Florida, Tampa.

## Data availability

The datasets used and/or analyzed during the current study are available from the corresponding author upon reasonable request.

## Funding

This work was supported by the US National Institutes on Aging grant AG057290.

## Conflict of competing interest

Gopal Thinakaran is an Associate Editor of *Genes & Diseases* and was not involved in the editorial review or the decision to publish this article. The authors declare that they have no other competing interests.
