# Supplementary material for: Amylin exacerbates tau pathology in the visual cortex of diabetic mice by impairing lysosomal activity
Source: Genes Dis. 2025 Mar 18;12(5):101602. doi: 10.1016/j.gendis.2025.101602 (PMC12221597; doi:10.1016/j.gendis.2025.101602)
Supplement: Multimedia component 1 [file mmc1.docx]

**List of antibodies used in this study**

| Primary Antibodies | Source | Catalog identifier | Dilution |
| --- | --- | --- | --- |
| Mouse monoclonal anti-AT8 | Invitrogen | MN1020 | 1:500 |
| Mouse monoclonal anti-MC1 | Peter Davies Lab |  | 1:500 |
| Rabbit polyclonal anti-NeuN | Sigma | ABN78 | 1:1000 |
| Mouse monoclonal anti-GFAP | Sigma | G3893 | 1:100 |
| Goat polyclonal anti-IBA1 | Abcam | ab5076 | 1:2000 |
| Rat monoclonal anti-LAMP1 | Developmental Studies Hybridoma | 1D4B | 1:200 |
| Rabbit monoclonal anti-CatD | Abcam | ab75852 | 1:1000 |
| Rabbit polyclonal anti-Amylin | Peninsula Lab | T-4157 | 1:1000 |
| Guinea pig polyclonal anti-Insulin | Abcam | ab7842 | 1:200 |
| **Secondary Antibodies** | **Source** | **Catalog identifier** | **Dilution** |
| Alexa Fluor 488 donkey anti-mouse | Invitrogen | A32766 | 1:250 |
| Alexa Fluor 555 donkey anti-mouse | Invitrogen | A31570 | 1:250 |
| Alexa Fluor 488 donkey anti-rabbit | Invitrogen | A21206 | 1:250 |
| Alexa Fluor 555 donkey anti-rabbit | Invitrogen | A31572 | 1:250 |
| Alexa Fluor 555 donkey anti-goat | Invitrogen | A11055 | 1:250 |
| Alexa Fluor 555 goat anti-guinea pig | Invitrogen | A21435 | 1:250 |
| Alexa Fluor 555 goat anti-rat | Invitrogen | A21434 | 1:250 |
